# Frame-shifted APOBEC3A encodes two alternative proapoptotic proteins that target the mitochondrial network

**DOI:** 10.1016/j.jbc.2021.101081

**Published:** 2021-08-14

**Authors:** Vincent Caval, Rodolphe Suspène, Pierre Khalfi, Julien Gaillard, Grégory Caignard, Damien Vitour, Philippe Roingeard, Jean-Pierre Vartanian, Simon Wain-Hobson

**Affiliations:** 1 Molecular Retrovirology Unit, Institut Pasteur, Paris, France; 2 Sorbonne Université, Complexité du Vivant, ED515, Paris, France; 3 Morphogenèse et Antigénicité du VIH et des Virus des Hépatites, Inserm-U1259 MAVIVH, Université de Tours and CHRU de Tours, Tours, France; 4 Plate-Forme IBiSA des Microscopies, PPF ASB, Université de Tours and CHRU de Tours, Tours, France; 5 UMR Virologie, INRAE, Ecole Nationale Vétérinaire d'Alfort, Laboratoire de santé animale d'Alfort, Anses, Université Paris-Est, Maisons-Alfort, France

**Keywords:** APOBEC3A, A3Alt, apoptosis, mitochondria, A3A-H, APOBEC3A-APOBEC3H, AIF, apoptosis-inducing factor, APE1/APE2, apurinic/apyrimidinic (AP) endonuclease 1/2, APOBEC3, Apolipoprotein B mRNA editing enzyme, catalytic polypeptide-like, ATXN1, ataxin 1, ATP, adenosine triphosphate, CCCP, carbonyl cyanide 3-chlorophenylhydrazone, cDNA, complementary DNA, DCFDA, 2′,7′-dichlorodihydrofluorescein diacetate, DMSO, dimethyl sulfoxide, DNA, deoxyribonucleic acid, DSB, double strand break, GFP, green fluorescent protein, GNAS, guanine nucleotide-binding protein, alpha stimulating, IFN, interferon, Kb, kilo base, Luc, luciferase, MEK1/2, MAP/ERK kinase-1/2, MOMP, mitochondrial outer membrane permeabilization, MPP, mitochondrial processing peptidase, mtDNA, mitochondrial DNA, mRNA, messenger RNA, nuDNA, nuclear DNA, PARP, Poly (ADP-ribose) polymerase, PBMC, peripheral blood mononuclear cell, PCR, polymerase chain reaction, PRNP, prion protein, RNA, ribonucleic acid, ROS, reactive oxygen species, RTqPCR, reverse transcription quantitative PCR, ssDN, single-stranded DNA, TBHP, tertiary-butyl hydroperoxide, TFAM, transcription factor A, mitochondrial, TOM20, translocase of outer membrane protein 20, TRIB3, Tribbles homolog 3, UNG, uracil-DNA glycosylase, UTR, untranslated region, γH2AX, phosphorylated histone H2AX

## Abstract

The human APOBEC3A (A3A) cytidine deaminase is a powerful DNA mutator enzyme recognized as a major source of somatic mutations in tumor cell genomes. However, there is a discrepancy between *APOBEC3A* mRNA levels after interferon stimulation in myeloid cells and A3A detection at the protein level. To understand this difference, we investigated the expression of two novel alternative “A3Alt” proteins encoded in the +1-shifted reading frame of the *APOBEC3A* gene. A3Alt-L and its shorter isoform A3Alt-S appear to be transmembrane proteins targeted to the mitochondrial compartment that induce membrane depolarization and apoptosis. Thus, the *APOBEC3A* gene represents a new example wherein a single gene encodes two proapoptotic proteins, A3A cytidine deaminases that target the genome and A3Alt proteins that target mitochondria.

The human APOBEC3 (A3) locus encodes six functional polynucleotide cytidine deaminases (A3A–C and A3F–H) (1), originally described as innate cellular defenses against retroviruses (2, 3, 4, 5), DNA viruses (6, 7, 8), and retroelements (9, 10, 11) mostly through cytidine deamination of single-stranded DNA (ssDNA). The mutagenic activity of APOBEC3A (A3A) and APOBEC3B (A3B) has recently been demonstrated to introduce somatic mutations in genomic DNA (12, 13), and those two enzymes are now recognized as endogenous causal agents responsible for the accumulation of CG to TA transitions in cancer genomes (14). As a result of cytidine deamination, inappropriate uracil bases in DNA can be removed by uracil N-glycosylase enzyme (UNG) and generate abasic sites, further processed by apurinic/apyrimidinic endonucleases, APE1 and APE2 resulting in double-strand break (DSB) formation and apoptosis (15, 16). It is now accepted that both A3A and A3B are intrinsic mutators of chromosomal DNA, although some debate persists regarding the contribution of each enzyme to the accumulation of mutations paving the way to cancer formation (17, 18, 19, 20, 21, 22). However, recent data revealing episodic waves of APOBEC3 (A3) mutations in various cancer genomes (21, 23) favor a scenario in which A3A plays the main role in oncogenesis, since this enzyme is known to be upregulated through interferon (IFN) signaling in response to many cellular stress (12, 24, 25, 26), further emphasizing the long-standing observation that cancer emerges on a background of chronic inflammation (27).

To limit nuclear DNA damage, *A3A* expression is controlled at multiple levels. If *A3A* promoter regulation remains so far elusive, with the exception of demonstrated IFN responsiveness in hematopoietic cells (24), *A3A* mRNA expression appears almost undetectable in other tissues. *A3A*-3′UTR also contributes to reducing A3A expression, since its substitution by *A3B*-3′UTR in the context of a prevalent *A3B* deletion allele (Δ*A3B*) (28) results in increased A3A levels and nuclear DNA (nuDNA) damages (13), in keeping with the overrepresentation of APOBEC mutations in the cancer genomes of Δ*A3B* patients (18). Ultimately, at the posttranslational level, the nuclear fraction of A3A has been demonstrated to be degraded by TRIB3 enzyme, reducing nuDNA editing and chromosomic DNA damage (29).

The human *A3A* mRNA is known to encode two functional isoforms of A3A cytidine deaminase (16), A3A-Long (A3A-L) and A3A-Short (A3A-S), that are functionally equivalent (16, 30). The initiating methionine codons governing the expression of A3A-L and A3A-S isoforms are only described as “adequate” in the language of Kozak (31, 32, 33, 34), explaining the submaximal detection of A3A cytidine deaminase at the protein level, whatever the degree of A3A mRNA upregulation upon stimulation (25). This configuration allows 40S ribosomal subunit to bypass those triplets and initiate translation at downstream start sites in a process called “leaky scanning” (31, 32, 33, 34).

In the present work, we report the identification of a novel ORF of 96 residues in the +1-shifted reading frame that completely overlaps that of *A3A*. Initiation from two methionine codons gives rise to two proteins, A3Alt-L (10.5 kDa) and the smaller A3Alt-S (8.6 kDa) that differ only at their N-termini by 18 residues. Functional characterization reveals that they specifically target mitochondria, resulting in mitochondrial membrane potential depolarization and apoptosis.

Thus *A3A* gene appears to be a new example of completely overlapping “dual coding genes,” which are rare in the human genome (35, 36, 37, 38, 39). At high levels A3A cytidine deaminases are genotoxic while the A3Alt proteins are toxic for the mitochondria. Individually both are proapoptotic. As the gene is stimulated by IFN and other forms of cellular stress, *A3A* appears to be a bifunctional stress-sensitive cytocidal gene.

## Results

### *APOBEC3A* gene encodes two novel alternative proteins, A3Alt-L and A3Alt-S

**Figure 1:**
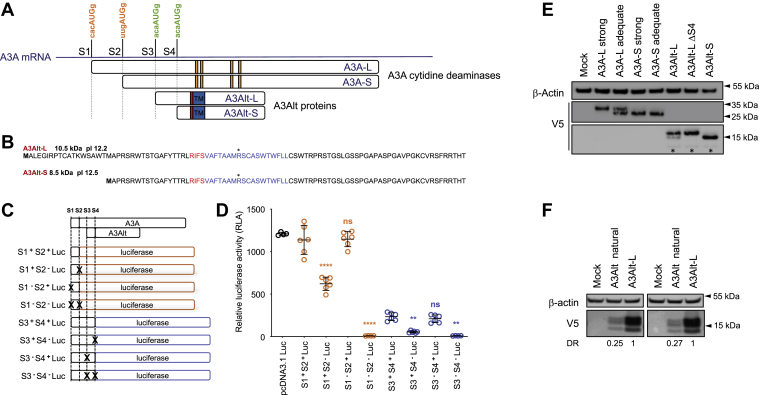
**A3A mRNA encodes novel A3Alt proteins.***A*, schematic representation of differential translation from A3A mRNA. A3A-L and A3A-S cytidine deaminases are translated from S1 and S2 “adequate” Kozak initiation codons (in *orange*). Catalytic residues involved in zinc coordination are depicted in *yellow*. A3Alt-L and A3Alt-S are translated from S3 and S4 “strong” Kozak initiation codons (in *green*). Mitochondrial addressing signal RIFS is highlighted in *red* and predicted transmembrane domain is shown in *blue*. *B*, A3Alt-L and A3Alt-S protein sequences. Mitochondrial addressing motif is shown in *red*, transmembrane domain is represented in *blue*. C, schematic representation of reporter constructs harboring 5′ sequence of A3A transcripts (in *white*) upstream of Firefly luciferase coding sequence cloned in A3A open reading frame (in *orange*) or in A3Alt +1 reading frame (in *blue*) to assess the differential translation initiation from start codons, S1, S2, S3, and S4. Start codons invalidated by site-directed mutagenesis are crossed out in *black*. *D*, firefly luciferase expression from S1, S2, S3, and S4 initiation codons, normalized on Renilla luciferase activity in HeLa cells 36 h after transfection. Error bars represent standard deviation from three independent transfections, measured in duplicates. Differences compared with S1^+^ S2^+^ Luc are represented in *orange*, and differences compared with S3^+^ S4^+^ are represented in *blue*. Differences were calculated using one-way ANOVA multiple comparison with Tukey post hoc test (ns: nonsignificant, ∗∗*p* < 0.002, ∗∗∗∗*p* < 0.0001). *E*, Western-blot analysis of V5-tagged A3A and A3Alt proteins in HeLa cells 24 h post transfection. β-Actin was used as loading control. Presented Western blot is representative of three independent experiments. *F*, Western blot analysis of A3Alt expression from A3A transcript in HeLa cells 24 h after A3Alt natural plasmid transfection where V5 tag is cloned in +1 reading frame at the end of A3Alt orf, in a plasmid containing A3A natural 5’ sequence. DR: Densitometric ratio (DR) representing A3Alt natural expression relative to A3Alt-L expression after normalization on β-Actin. Presented results correspond to two independent transfections.

The AUGs initiation codons S1 and S2 respectively governing the expression of A3A-L and A3A-S cytidine deaminases in *A3A* mRNA are only present in an “adequate” Kozak context, compatible with leaky scanning. We therefore sought for other downstream AUG codons in *A3A* classical transcripts (NM_145699.4, ENST00000249116, ENST00000618553). We identified two AUG (S3 and S4) in the +1 shifted reading frame of A3A, both of which being in a strong Kozak context (Figs. 1*A* and S1*A*). S3 defines an ORF of 96 residues encoding a small putative protein A3Alt-L (10.5 kDa, pI = 12.2), while the S4 methionine can be used in some alternatively spliced transcripts of *A3A* (NM_001270406.1), giving rise to an even smaller protein, A3Alt-S, of 78 residues (8.6 kDa, pI = 12.5) (Fig. 1, *A* and *B* and S1*A*). A3Alt-L and A3Alt-S only differ by 18 N-terminal residues. The observation was further confirmed by *in silico* analysis using the OpenProt tool, designed to unravel cryptic proteins encoded within transcripts (40), predicting A3Alt proteins translation from A3A mRNAs (Fig. S1, *B* and *C*).

To experimentally evaluate the differential translation from those four initiation codons, reporter constructs were generated fusing Firefly luciferase cDNA downstream of *A3A* 5′ sequence, either in A3A or in A3Alt + 1-shifted reading frames (Fig. 1*C*). AUG initiation codons were substituted by ACG by site-directed mutagenesis to estimate their relative contribution to protein expression (Fig. 1*C*). Reporter constructs were then transfected in HeLa cells along with a Renilla luciferase coding plasmid used as transfection control. After normalization to Renilla luciferase luminescence, quantitative Firefly detection revealed that Firefly luciferase expression driven by the combination of A3A initiation codons (S1 and S2, S1^+^ S2^+^ Luc) was on a par with the positive pcDNA3.1 Luc control (Fig. 1*D*). Unexpectedly in *A3A* S2 AUG (S1^−^ S2^+^ Luc) initiated a stronger expression than S1 alone (S1^+^ S2^−^ Luc) (Fig. 1*D*).

Analysis of initiation of A3Alt translation showed expression of ∼20% from A3Alt S3 and S4 (S3^+^ S4^+^ Luc) compared with A3A (S1^+^ S2^+^ Luc, Fig. 1*D*). Of the two potential A3Alt codons, S4 appears to be dominant. This indicates that both A3Alt proteins, A3Alt-L and A3Alt-S, can be expressed from A3A mRNA. *In vivo*, expression may be more complex, involving mRNA secondary structures as well as cis regulation by UTRs.

To explore the functionality of A3Alt proteins, A3Alt-L and A3Alt-S coding sequences were cloned in a V5-tag expression vector and transfected along with A3A-L and A3A-S cytidine deaminases expression plasmids in HeLa cells (schematic representation of V5 tagged constructs with Kozak context and stop codon positions is available in Fig. S2). In keeping with previous studies, A3A cytidine deaminase coding sequence driven by its natural “adequate” Kozak sequence (A3A-L adequate) resulted in the translation of the two functionally active isoforms A3A-L and A3A-S, while only A3A-L was detectable when cloned downstream of the strong Kozak initiation context of pcDNA3.1 expression plasmid (A3A-L strong) (Fig. 1*E*). Accordingly, the A3A-S coding plasmid, only containing the coding sequence of the shorter A3A-S isoform, allowed the detection of A3A-S isoform independently of the AUG Kozak context (A3A-S adequate or A3A-S strong) (Fig. 1*E*).

Transfection of A3Alt-L and A3Alt-S coding plasmids revealed suitable expression of both proteins, although A3Alt proteins levels were consistently lower than those obtained with A3A-L and A3A-S coding plasmids (Fig. 1*E*). Unexpectedly, although A3Alt-L translation in A3Alt-L expression plasmid was driven by a strong initiation codon, Western blot detection after A3Alt-L transfection revealed the unexpected expression of a shorter isoform migrating as A3Alt-S, confirming the finding obtained with luciferase reporter constructs that A3Alt-S S4 AUG is associated with a strong translational activity (Fig. 1*D*). Accordingly, S4 initiation codon was removed by mutagenesis from A3Alt-L expression plasmid to generate the A3A-L ΔS4 construct. Western blot probing of A3Alt-L ΔS4 transfection only revealed a single band at A3Alt-L expected size and therefore allowed the analysis of “full-length” A3Alt-L protein for functional study.

To validate A3Alt-L translation from A3A transcripts (Fig. 1*D*), an “A3Alt natural” construct was also generated corresponding to the A3A adequate construct, driven by its natural Kozak initiation context with terminal V5 tag cloned in the +1-shifted reading frame at the end of A3Alt coding sequence. Consistently with luciferase reporter transfection experiments, V5 probing confirmed A3Alt proteins expression from A3A transcript, albeit with reduced expression (∼25%) compared with A3Alt-L expression plasmid transfection (Fig. 1*F*).

### A3Alt and A3Alt-S are mitochondrial proteins

To help determine the functionality of those proteins, *in silico* analysis was conducted. Topology prediction was first performed using PSIPRED program (41) and both sequences were predicted to contain a central 21 amino acid transmembrane domain (Fig. 1, *A* and *B* in blue and Fig. S3*A* in pink). Subcellular localization prediction was next conducted using DeepLoc deep learning algorithms (42), locating A3Alt proteins to the mitochondria (Fig. S3*B*). Mitochondrial addressing was further explored by motif search revealing that the A3Alt N-terminus may act as a mitochondrial targeting peptide, potentially cleaved by Mitochondrial Processing Peptidase (MPP) through an R3a/R3b mitochondrial cleavage site motif (RIFS) identified using TPpred2 software (Fig. S3*C*) (43, 44). The identification of this putative MPP cleavage site that is present in both A3Alt-L and A3Alt-S proteins may be responsible for the smaller peptide detection evidenced in Western blot after A3Alt plasmids transfections (Fig. 1*E*, annotated with asterisks), potentially corresponding to the mature form of A3Alt proteins. Ultimately, this suggests that after cleavage both proteins may generate the same mature peptide and therefore exhibit the same functionality.

**Figure 2:**
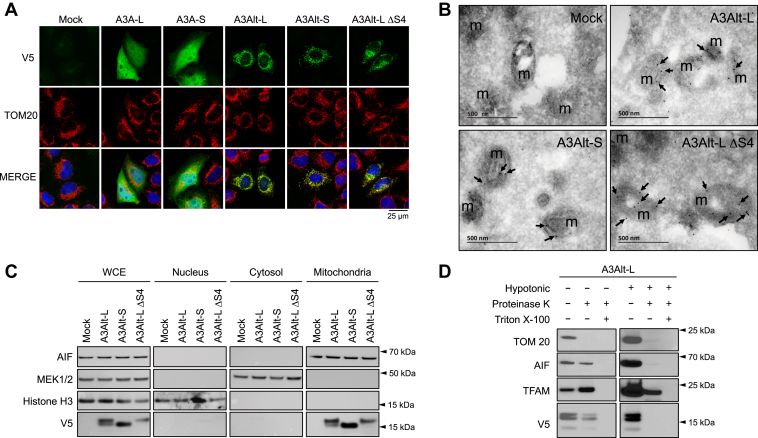
**A3Alt proteins are imported to the mitochondrial compartment.***A*, confocal microscopy of V5 tagged proteins in HeLa cells 24 h post transfection (in *green*). Nuclei are stained using DAPI (in *blue*) and mitochondrial compartment is evidenced using specific TOM20 antibody (in *red*). *B*, representative picture of cryoelectron microscopy detection of Flag tagged A3Alt proteins by immunogold staining in HeLa cells 24 h post transfection. *Black arrows* indicate representative immunogold staining, m the mitochondria. *C*, cellular fractions of HeLa transfected cells 24 h post transfection. V5 tag is used to detect A3Alt proteins, MEK1/2 is used as cytosolic marker, AIF as mitochondrial marker and Histone H3 as nuclear marker. Presented Western blots are representative of three independent experiments. *D*, Western blot analysis of proteinase K treatment of A3Alt-L transfected HeLa cells mitochondrial fraction resuspended in either isolation buffer (*left panel*) or hypotonic buffer (*right panel*). TOM20, AIF, and TFAM are markers for the mitochondrial external membrane, intermembrane space, and matrix, respectively. Triton X-100 detergent treatment was used as positive proteinase K digestion control. Presented western blots are representative of two independent experiments. WCE, whole cell extract.

To experimentally address mitochondrial localization of A3Alt proteins, HeLa cells were transfected with A3A and A3Alt expression plasmids and analyzed by confocal microscopy using TOM20 (Translocase of Outer Membrane protein 20) staining as mitochondrial marker (Fig. 2*A*). As previously reported, both A3A-L and A3A-S exhibited diffuse nucleocytoplasmic distribution, with no colocalization with mitochondrial compartment (12). By contrast, A3Alt-L, A3Alt-S, and full-length A3Alt-L ΔS4 consistently demonstrated a strong mitochondrial addressing, evidenced by TOM20 colocalization (Figs. 2*A* and S4 for lower magnification). Subcellular localization was further validated by cryo-electron microscopy and immunogold staining of Flag tagged A3Alt constructs, confirming mitochondrial addressing (Figs. 2*B* and S5 for Flag tagged A3Alt protein expression analysis). The transmembrane domain is somewhat unusual in that it encodes an arginine residue (asterisk Fig. 1*B*). However, alanine substitution in A3Alt-L R48A mutant construct did not alter addressing to the mitochondrial network (Figs. S6 and S4).

Subcellular fractionation was performed in transfected HeLa cells, confirming the localization of A3Alt proteins in apoptosis-inducing factor (AIF) positive mitochondrial fraction (Fig. 2*C*). In keeping with this, no V5 detection was found in the MEK1/2 positive cytosolic fraction, or the histone H3 nuclear fraction, confirming the specificity of A3Alt mitochondrial localization (Fig. 2*C*).

To further determine the submitochondrial compartmentalization of A3Alt proteins, crude mitochondria isolated from A3Alt-L transfected HeLa cells were isolated and submitted to proteinase K digestion with or without hypotonic treatment used to disrupt the mitochondrial outer membranes. While intact mitochondria treatment with proteinase K treatment resulted in complete TOM20 digestion, A3Alt-L protein detection using V5 probing was not impacted, suggesting that A3Alt proteins are not exposed on the outer face of the outer membrane the of mitochondria (Fig. 2*D*, left panel). Following hypotonic treatment, AIF protein localized in the intermembrane space was further degraded while the matrix associated protein TFAM was preserved from proteinase K digestion. In hypotonic condition, A3Alt-L detection was lost following proteinase K digestion suggesting that A3Alt proteins are located to the intermembrane space of transfected cells (Fig. 2*D*, right panel). The efficiency of proteinase K digestion was validated pretreating mitochondria with Triton X-100 detergent, resulting in the total loss of TOM20, A3Alt-L, AIF, and TFAM detection (Fig. 2*D*).

### A3Alt proteins induce mitochondrial outer membrane depolarization and apoptosis

**Figure 3:**
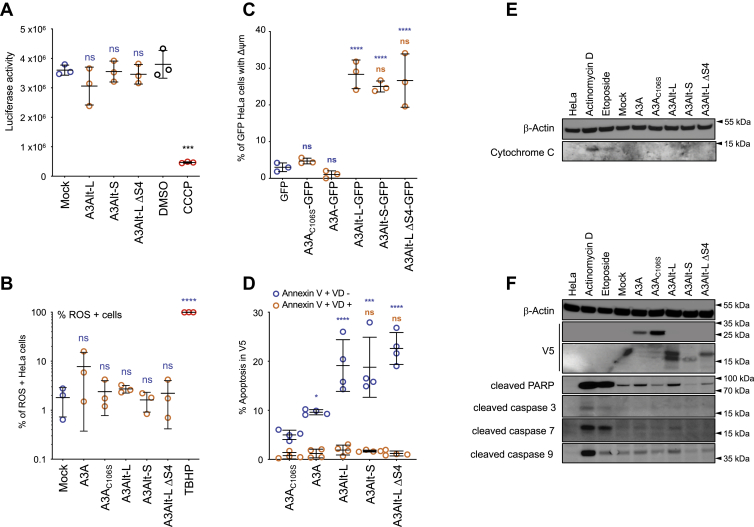
**A3Alt expression induces mitochondrial outer membrane depolarization and apoptosis.***A*, luciferase activity in A3A and A3Alt transfected HeLa cells cultivated in galactose supplemented glucose-free media. Results are from three independent transfections measured in duplicate. Treatment with carbonyl cyanide 3-chlorophenylhydrazone (CCCP), a mitochondrial oxidative phosphorylation uncoupler, was used as positive control. Differences compared with mock transfected cells are represented in *blue*, difference compared with DMSO-treated HeLa cell are represented in *black*. Differences were calculated using one-way ANOVA multiple comparison with Tukey post hoc test (ns: nonsignificant, ∗∗∗*p* < 0.001). *B*, Reactive Oxygen Species (ROS) levels assessed by DCFAD staining in A3A and A3Alt transfected HeLa cells. Oxidative stress inducer TBHP (100 μM) was used as positive control for ROS production. Data are from three independent transfections, and differences were calculated using one-way ANOVA multiple comparison with Tukey post hoc test (ns: nonsignificant, ∗∗∗∗*p* < 0.0001). *C*, flow cytometry analysis of mitochondrial membrane potential depolarization (Δψm) after with MitoStatus Red specific dye staining in HeLa cells transfected with GFP-tagged A3A and A3Alt expression plasmids from three independent transfections. Differences compared with GFP transfected cells are represented *blue*, and differences compared with A3Alt-L-GFP transfected cells are represented in *orange*. Differences were calculated using one-way ANOVA multiple comparison with Tukey post hoc test (ns: nonsignificant, ∗∗∗∗*p* < 0.0001). *D*, flow cytometry analysis of cellular death in transfected HeLa cells. Annexin V +, VD-cells are represented in *blue*, Annexin V +, VD cells + in *orange*. Error bars represent standard deviation from four independent transfections. Differences compared with A3A_C106S_ catalytic mutants are represented *blue*, and differences compared with A3Alt-L are represented in *orange*. Differences were calculated using one-way ANOVA multiple comparison with Tukey post hoc test (ns: nonsignificant, ∗*p* < 0.05, ∗∗∗*p* < 0.002, ∗∗∗*p* < 0.0001). *E*, Western blot analysis of cytoplasmic cytochrome C release in cytoplasmic fraction of A3A and A3Alt transfected HeLa cells. β-Actin was used as loading control. Presented western blots are representative of three independent experiments. *F*, Western blot analysis of cleaved caspase pathway following A3A and A3Alt expression in HeLa cells, 24 h after transfection. β-Actin was used as loading control. HeLa cells treated for 16 h with 100 μM etoposide or 100 μM actinomycin D were used as positive controls. Presented Western blots are representative of three independent experiments.

Mitochondria play a central role in the life and death of eukaryotic cells. We thus investigated whether A3Alt expression had an effect on mitochondrial respiration and ATP production. Therefore, HeLa cells cultivated in galactose supplemented glucose-free media were transfected with A3Alt expression plasmids and ATP production was evaluated measuring luciferase activity. Quantitative luminescence revealed that none of A3Alt construct significantly impacted ATP production compared with mock transfected cells (Fig. 3*A*). Treatment with carbonyl cyanide 3-chlorophenylhydrazone (CCCP), a mitochondrial oxidative phosphorylation uncoupler, was used as positive control and resulted a significative drop in luciferase activity compared with DMSO-treated HeLa cells (Fig. 3*A*). As A3A cytidine deaminases genotoxicity has recently been associated with reactive oxygen species (ROS) accumulation (45) in an experimental setting compatible with A3Alt proteins expression, we next explored whether they could dysregulate ROS production at the mitochondrial level. Accordingly, A3A and A3Alt expression plasmids were transfected in HeLa cells and incubated with 2′,7′-dichlorodihydrofluorescein diacetate (DCFDA) dye, commonly used to measure ROS production as its fluorescence is proportional to DCFDA oxidation. None of the constructs were associated with significant ROS accumulation, which was only found using tertiary-butyl hydroperoxide TBHP oxidative stress inducer (Figs. 3*B*, and S7). However, although A3A expression been previously described to induce ROS production (45), only a modest increase of A3A induced ROS production (∼8%) that did not score as significant compared with mock-transfected cells was observed in our experimental setup, which was not recapitulated with A3A_C106S_ catalytic mutant (∼2.4%) (Figs. 3*B* and S7).

We next sought to investigate if A3Alt proteins could impact mitochondrial outer membrane potential, which can reflect the initiating event of mitochondrial outer membrane permeabilization (MOMP) and intrinsic mitochondrial apoptosis pathway. The loss of inner mitochondrial membrane potential (Δψm) was evaluated using MitoStatus Red specific dye in transfected HeLa cells. Since mitochondrial membrane potential staining is not compatible with fixation and permeabilization, functional C-terminally GFP-tagged constructs were generated to allow gating on A3A and A3Alt expressing cells (Fig. S8). If A3A-GFP and A3A_C106S_-GFP failed to modify mitochondrial potential compared with GFP control transfection, A3Alt-L-GFP, A3Alt-S-GFP, and A3Alt-L ΔS4-GFP consistently elicited mitochondrial membrane potential depolarization, arguing for mitochondrial outer membrane permeabilization (Figs. 3*C* and S9). Cell death was therefore assessed using Annexin V probing and viability dye staining to detect early and late apoptosis on transfected V5 positive cells. Etoposide-treated HeLa cells were used as positive apoptosis staining control (Fig. S10). As previously described, A3A transfection induced significative levels of apoptosis (∼10%) compared with the catalytically inactive A3A_C106S_ mutant (∼4%) (Fig. 3*D*). Strikingly A3Alt-L, A3Alt-S as well as A3Alt-L ΔS4 transfections resulted in strong and consistent cellular death (∼22%) (Figs. 3*D* and S10). In keeping with what was inferred from *in silico* analysis, all A3Alt proteins exhibited analogous proapoptotic activity.

However, cytochrome C release analysis by Western blot probing of cytosolic extracts failed to evidence cytochrome C protein in the cytoplasm of A3A and A3Alt transfected cells (Fig. 3*E*). In keeping with this observation, caspase activation pathway analysis using Western blot probing of cleaved PARP and cleaved caspase 3, 7, and 9 after A3A and A3Alt transfection failed to demonstrate a clear activation of caspase-dependent apoptosis pathway beyond the levels observed with mock-transfected cells (Fig. 3*F*) as it was previously observed following A3A expression (16). This finding indicates that although A3Alt expression perturbs inner mitochondrial membrane potential (Fig. 3*C*), it might not result in outer membrane permeabilization associated with cytochrome C release and canonical caspase activation pathway. Accordingly, A3Alt-L transfected HeLa cells treatment with Z-VAD-FKM pan-caspase inhibitor did not significantly reduce A3Alt-induced apoptosis (Fig. S11), while etoposide-induced cell death was totally abrogated (Fig. S11). Altogether, these data indicate that A3Alt proteins are addressed to the mitochondrial network and elicit cellular death, in a caspase-independent process.

### A3A proapoptotic nature is independent of A3Alt orf

**Figure 4:**
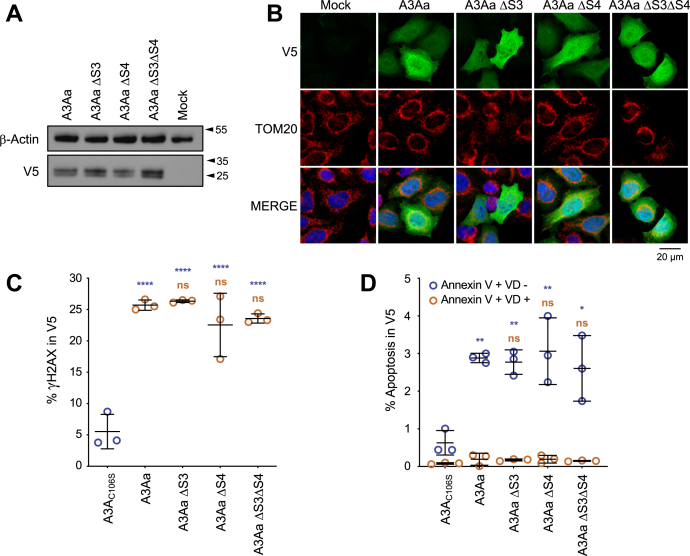
**A3A induces apoptosis independently of A3Alt expression.***A*, Western blot analysis of V5-tagged A3Aa, possessing A3A natural adequate initiation codon, and mutants devoid of internal S3 and S3 AUGs, in HeLa cells 24 h post transfection. β-Actin was used as loading control. Presented Western blots are representative of three independent experiments. *B*, confocal microscopy of V5 tagged A3Aa mutants in HeLa cells 24 h post transfection (in *green*). Nuclei are stained using DAPI (in *blue*) and mitochondrial compartment is evidenced using specific TOM20 antibody (in *red*). *C*, flow cytometry analysis of γH2AX-positive HeLa cells gated on V5-positive cells after A3Aa and A3Aa mutant transfections after 48 h. Error bars represent standard deviation from three independent transfections. Differences compared with A3A_C106S_ catalytic mutants are represented in *blue*, differences compared with A3Aa are represented in *orange*. Differences were calculated using one-way ANOVA multiple comparison with Tukey post hoc test (ns: non -significant, ∗∗∗∗*p* < 0.0001). *D*, flow cytometry analysis of cellular death in transfected HeLa cells. Annexin V +, VD-cells are represented in *blue*, Annexin V +, VD cells + in *orange*. Error bars represent standard deviation from three independent transfections. Differences compared with A3A_C106S_ catalytic mutant are represented *blue*, and differences compared with A3Aa are represented in *orange*. Differences were calculated using one-way ANOVA multiple comparison with Tukey post hoc test (ns: nonsignificant, ∗*p* < 0.05, ∗∗*p* < 0.005).

To assess the implication of A3Alt translation in A3A proapoptotic activity, mutagenesis was performed in A3A-L adequate (A3Aa) coding plasmid to remove S3 and S4 initiation codons without changing the A3A protein sequence (Fig. S2). Upon transfection, A3Aa ΔS3, A3Aa ΔS4, and A3Aa ΔS3ΔS4 resulted in similar levels of A3A proteins (Fig. 4*A*), displaying typical pancellular localization (Figs. 4*B* and S4). All constructions demonstrated similar functionality in DSB formation, assessed by quantification of histone H2AX phosphorylation (γH2AX) in V5 positive cells (∼25% γH2AX) (Figs. 4*C* and S12), compared with A3A_C106S_ negative control (∼6% γH2AX). Apoptosis was evaluated and showed that every construct equally induced apoptosis (∼6%) compared with A3A catalytic mutant (∼1.5%), confirming that A3A cytidine deaminase genotoxic activity is by itself capable of inducing apoptosis (Figs. 4*D* and S12), albeit at lower level than A3Alt proteins (Fig. 3*D*).

## Discussion

In the present work, we report that human *A3A* gene encodes two distinct proteins. A3A cytidine deaminase, which can exist as two functional isoforms A3A-L and A3A-S (16), is a highly versatile enzyme involved in innate immune responses against many viruses (2, 3, 4, 5, 6, 7, 8) and retroelements (9, 10, 11), as well as in nucleic acid catabolism to evade self-DNA sponsored inflammation (25). However, the price to pay for this mutator enzyme activity is off-target editing associated with somatic mutations and chromosomal rearrangements in many cancer genomes (14, 21, 23, 46, 47). To date, no deletion or inactivation allele has been reported for A3A unlike other APOBEC3 family members (28, 48). Accordingly, understanding the regulation of A3A expression is now of major concern, as its expression has also been reported to exert additional effects on cellular physiology, causing cell cycle arrest, apoptosis, as well as tumorigenic ROS production (15, 16, 45).

In trying to understand the observed discrepancy between *A3A* mRNA levels after stimulation in myeloid cells and A3A detection at the protein level (25), we identified an out-of-frame overlapping reading frame in A3A transcript, predicted to encode novel small alternative A3Alt proteins, A3Alt-L, and the shorter isoform A3Alt-S. If we failed to generate A3Alt specific antibodies immunizing rabbits with peptides corresponding to A3Alt-L N-terminal et C-terminal fragments, which would have allowed *in vivo* probing of A3Alt peptides in cells such as THP1 or isolated PBMCs known to express high level of *A3A* mRNA upon stimulation, the biological relevance of A3Alt is grounded in several observations. Analysis of orthologous *A3A* genes in primates revealed that the atypical organization AUG initiation codons in human *A3A* gene is shared among great apes, with A3A cytidine deaminases expression driven by “weak” AUGs while A3Alt orfs are present in a favored expression Kozak context (Fig. S13). Thus, the A3Alt reading frame is conserved in many primates, arguing for functionality. A3Alt expression *in vivo* is also further supported by mass spectrometry analysis, identifying A3Alt-L-derived peptide MALEGIRPTCATKWSAWTMAPR in the Bioplex 2.0 wide scale interactome study (49), which corresponds to the 22 N-terminal residues of A3Alt-L (Fig. S1*B*). This singular configuration, a single gene encoding two distinct proteins is not unprecedented with a handful of notable examples such as ATXN1 (39), GNAS complex locus (36, 37), prion-related PRNP (38) as well as INK4α tumor suppressor gene (35).

*In silico* analysis validated by functional study revealed that those new A3Alt proteins were targeted to the mitochondrial compartment, resulting in inner mitochondrial membrane potential depolarization as well as apoptosis. However, if Annexin V staining was demonstrated in V5 transfected cells using flow cytometry, we failed to demonstrate global cytochrome C release as well as caspase activation upon A3Alt coding plasmid transfection. This may suggest that either the cell death pathway involved is independent of canonical apoptosis program or that our experimental setup, relying on plasmid transfection, may be suboptimal to study into details the mechanism involved in A3Alt-mediated cell death. Unfortunately, every attempt to generate stable cell lines expressing A3Alt, either by lentiviral transduction or plasmid-based expression system (Tetracycline-Regulated Expression T-Rex System, Thermo Fisher Scientific), proved unsuccessful, probably given the proapoptotic nature of our proteins. The same is true for high-level inducible A3A expressing cell lines.

Be that as it may, the presented results culminate in a singular situation where two proapoptotic proteins, A3A cytidine deaminases targeting the genome, and A3Alt proteins targeting the mitochondrion, are encoded by a single gene. As A3A genotoxicity is reported to mainly affect replicating cells where nuclear genome is exposed as ssDNA and vulnerable to A3A mutational activity (50), A3Alt-mediated apoptosis may represent an alternative way to induce cellular death in overstimulated quiescent cells.

Interestingly, this study also tightens the emerging links between A3A expression and mitochondrial network homeostasis, as A3A has recently been reported to be central in cytoplasmic mitochondrial DNA (mtDNA) catabolism (25, 26), to prevent danger signal accumulation and inflammation (51). The impact of A3Alt on mtDNA release into the cytoplasm will therefore need to be evaluated to better understand the contribution of A3A and A3Alt in balancing the cytoplasmic mtDNA level in cells following cellular stress. One can imagine a scenario in which modest stimulation will only result in A3A cytidine deaminase expression and agonist mtDNA clearance (25, 26), while excessive stress may result in A3A and A3Alt expression, where A3Alt sponsored mitochondrial alterations may fuel mtDNA release to the cytosol and in return overstimulate *A3A* gene expression, leading to cell death. This observation emphasizes the complexity of *A3A* gene regulation, which stands at the crossroads of prosurvival interferon signaling pathways, and two distinct mechanisms leading to cell death.

In conclusion, it is intriguing that among the few examples of “dual-coding genes” A3A, an innate immunity related gene shaped by a long-standing coevolution process with pathogens, appears to have appropriated a mechanism that is common for many viruses and bacteria. More intriguingly, some proteins from overlapping reading frames from Chicken Anemia Virus, Influenza A Virus, and some Norovirus are involved in pathogenicity by targeting the mitochondria, culminating in cell death (52, 53, 54).

## Experimental procedures

### Plasmids

A3A-L strong (A3A-L), A3A-L adequate (A3Aa), A3A-S strong (A3A-S), A3A-S adequate, and catalytic mutants have already been described (16). A3A, A3Alt, and derivatives were generated by PCR (Table S1) and cloned into pcDNA3.1D/V5-His-TOPO vector (Thermo Fisher Scientific). A3Alt-L ΔS4, A3Aa ΔS3, A3Aa ΔS4, A3Aa ΔS3ΔS4, and A3Alt-L R48A were generated by site-directed mutagenesis (GeneArt Site-Directed Mutagenesis System, Thermo Fisher Scientific, Table S2). GFP-tagged plasmids were constructed upon amplification of GFP coding sequence using primers designed to add NotI/XbaI restriction site NotI-GFPfwd: TTGCGGCCGCATGGTGAGCAAGGGCGAGGAGC, XbaI-GFPrev: TTTCTAGATTGTACAGCTCGTCCATGCCG, and amplicon was in-frame inserted using NotI/XbaI cloning in pcDNA3.1D/V5-His-TOPO vector C-terminal linker. APOBEC3A-luciferase coding plasmids were constructed by PCR and cloned into pcDNA3.1D/V5-His-TOPO vector (Thermo Fisher Scientific). All constructs were grown in *E. coli* TOP10 cells (Thermo Fisher Scientific) and verified by sequencing.

### Cells

Human HeLa cells were maintained in DMEM glutamax medium (Thermo Fisher Scientific) supplemented with 10% FCS, 50 U/ml penicillin, and 50 mg/ml streptomycin.

### Transfections

Plasmid transfection was performed on 8 × 10^5^ of HeLa cells, transfected using 2 μg of expression plasmids using Fugene HD (Roche) following manufacturer’s recommendations and harvested 24 h posttransfection. For immunofluorescence labeling, 5 × 10^4^ HeLa cells grown on chamber slides (LabTek) were transfected with 1 μg of expression plasmids using Fugene HD (Roche) following manufacturer’s recommendations.

### Luciferase activity

For luciferase activity assay, 8 × 10^5^ of HeLa cells grown in six wells plates were cotransfected with 1.8 μg of firefly luciferase constructs along with 0.2 μg of Renilla luciferase control plasmid (Promega) using Fugene HD (Roche) following manufacturer’s recommendations. Transfected cells were split in 96-well at a density of 5 × 10^4^ cells/well, and luciferase activity was measured 24 h later using Dual-Glo luciferase assay (Promega) with 30 min incubation times. Presented results represent luciferase activity from three independent transfections measured in triplicates.

### Western blotting

Harvested transfected cells were submitted to freeze/thaw lysis cycle in RIPA lysis buffer (25 mM Tris-HCl pH7.6, 150 mM NaCl, 1% NP-40, 1% sodium deoxycholate, 0.1% SDS) supplemented with Complete Protease Inhibitor Mixture (Roche Applied Science). Cell lysates were then clarified by centrifugation at 14,000*g* for 30 min. Western blot analysis on cell lystates was carried out according to standard procedures. V5 probing was performed using 1:5000 diluted horseradish peroxidase–coupled mouse monoclonal antibody (R961–25, Thermo Fisher Scientific) in PSB-0.1% Tween 5% dry milk. Flag tag probing was performed using 1:5000 diluted horseradish peroxidase–coupled mouse monoclonal antibody (clone M2, A8592, Sigma) in PSB-0.1% Tween 5% dry milk. β-Actin probing was performed using 1:50,000 diluted horseradish peroxidase–coupled monoclonal antibody (clone AC-15, A3854, Sigma) in PSB-0.1% Tween 5% dry milk. GFP probing was performed using 1:2500 diluted mouse monoclonal antibody (clone 4B10B2, MA5-15349, Thermo Fisher Scientific) in PSB-0.1% Tween 5% dry milk followed by washings and incubation with an anti-mouse IgG horseradish peroxidase–coupled secondary antibody (NA931V, GE Healthcare). Cytochrome C antibody, cleaved caspase pathway antibodies (cleaved caspase 3, 7, 9 and cleaved PARP) and cell fractionation specific antibodies (AIF, MEK1/2, Histone H3) were produced in rabbit (#4272, #9929, #11843, Cell Signaling Technology) and used 1:1000 in TSB-0.1% Tween 5% bovine serum albumin. After washings, membranes were probed with anti-rabbit IgG horseradish peroxidase–coupled secondary antibody (#7074, Cell Signaling Technology) diluted 1:5000 in TSB-0.1% Tween 5% bovine serum albumin. Membrane was revealed by enhanced chemiluminescence substrates (Pierce). Uncropped versions of Western blots are provided in Figure S14.

### Subcellular fractionation

At 24 h, six million HeLa transfected cells were recovered and washed in cold PBS. One million cells were collected and lysed in RIPA lysis buffer for western blotting the whole-cell extract (WCE). Subcellular fraction was then performed on the rest of the cells adapting a previously published protocol (55). Briefly, HeLa cells were resuspended in digitonin buffer (150 mM NaCl, 50 mM HEPES pH7.4 and 100 μg/ml of digitonin) for 10 min under gentle agitation. Lysates were then centrifuged for 10 min at 2000*g* to pellet nuclei and organelles. Supernatants were centrifuged three times at 20,000*g* for 20 min to purify cytosolic fraction. Nuclei and organelles pellets were washed twice in cold PBS and resuspended in NP40 lysis Buffer (150 mM NaCl, 50 mM HEPES pH7.4, 1% NP-40, protease, and phosphatase inhibitors) and incubated for 30 min on ice. Lysates were then centrifuged for 10 min at 7000*g*. Supernatants corresponding to the mitochondrial fraction were collected. Pellets were washed twice in PBS, resuspended in RIPA lysis buffer, and sonicated.

### Subcellular fractionation and proteinase K treatment

Transfected HeLa cells were collected, washed two times in PBS, and washed one time in isolation buffer (10 mM HEPES-KOH, pH 7.4, 0.25 M sucrose, and 1 mM EDTA). Cells were then homogenized on ice using Potter-Elvehjem homogenizer (20 strokes) and centrifuged at 900*g* for 10 min to pellet nuclear fraction and intact cells. The supernatant was collected and centrifuged at 10,000*g* for 10 min. The second pellet corresponding to the crude mitochondrial fraction was resuspended either in isolation buffer or hypotonic buffer (10 mM HEPES-KOH, pH 7.4, and 1 mM EDTA) and incubated on ice for 10 min to allow mitochondrial outer membranes disruption. Mitochondria were then digested with proteinase K at a final concentration of 100 μg/ml, with or without 1% Triton X-100 on ice for 15 min. Reaction stopped by adding the protease inhibitor PMSF at a final concentration of 1 mM for 15 min on ice, and samples were resuspended in reducing 4× LDS sample buffer (ThermoFisher Scientific).

Mitochondrial proteins were probed with anti-AIF monoclonal rabbit antibody (#4272, Cell Signaling Technology), anti-TOM20 rabbit monoclonal antibody (ab186734, Abcam), and anti-TFAM monoclonal mouse antibody (18G102B2E11, Thermo Fisher Scientific) used 1:1000 in TSB-0.1% Tween 5% bovine serum albumin and incubated overnight at 4 °C. After washings, membranes were probed with anti-rabbit IgG horseradish peroxidase–coupled secondary antibody (#7074, Cell Signaling Technology) or anti-mouse IgG horseradish peroxidase–coupled secondary antibody (NA931-1ML, Amersham) diluted 1:5000 in TBS-0.1% Tween 5% bovine serum albumin. Membrane was revealed by enhanced chemiluminescence substrates (Pierce).

### Immunofluorescence

After PBS washings, transfected HeLa cells grown on chamber slides were fixed with 4% PFA for 15 min. After PBS washing, cells were incubated in 50/50 acetone/methanol for 20 min. Mouse monoclonal anti-V5 antibody (R960-25, Thermo Fisher Scientific), mouse monoclonal anti-Flag antibody (clone M2, F1804, Sigma), mouse monoclonal anti-GFP antibody (clone 4B10B2, MA5-15349, Thermo Fisher Scientific), and rabbit monoclonal anti-TOM20 (ab186734, Abcam) were incubated at 1/250 for 1 h at room temperature, followed by incubation with a mouse specific Alexa-488 goat antibody (Thermo Fisher Scientific) and rabbit specific alexa-633 conjugated donkey antibody (Thermo Fisher Scientific) 1 h at room temperature in the dark. After washing, slides were mounted with Fluoromount-G imaging medium containing DAPI (Sigma). Imaging was performed using Leica SP5 confocal microscope.

### Immunogold labeling of cryosections for immunoelectron microscopy

Cells were fixed for 2 h with 4% paraformaldehyde/0.1% glutaraldehyde in phosphate buffer (pH7.6), washed with PBS (pH7.6) for 2 × 5 min, and centrifuged at 500*g* for 10 min. After removing the supernatant, cell pellets were included in gelatin 12% and infused with sucrose 2.3 M overnight at 4 °C. 90 nm ultrathin cryosections were made a −110 °C on a LEICA UCT cryo-ultramicrotome. Sections were retrieved with Methylcellulose 2%/Sucrose 2.3 M mixture (1:1) and collected onto formvar/carbon-coated nickel grids. After removal of gelatine at 37 °C, sections were incubated on drops of 1:100 anti-Flag (F7425, Sigma). After six washes of 5 min each, grids were incubated on drops of PBS containing 1:30 gold-conjugated (10 nm) goat-anti-rabbit IgG (Aurion). Grids were finally washed with six drops of PBS (5 min each), postfixed in 1% glutaraldehyde, and rinsed with three drops of distilled water. Contrasting step was performed by incubating grids on drops of uranyl acetate 2%/methylcellulose 2% mixture (1:10). The sections were imaged in a transmission electron microscope at 100 kV (JEOL 1011).

### ATP production analysis

HeLa cells were cultivated in glucose-free DMEM medium (Thermo Fisher Scientific) supplemented with 10% FCS, 50 U/ml penicillin, 50 mg/ml streptomycin, and 10 mM galactose (Sigma). Plasmid transfection was performed as previously described and culture media was replaced with serum-free glucose-free DMEM medium 6 h after transfection. Transfected cells were harvested 24 h after transfection and split in 96-well plate at a density of 5 × 10^4^ cells/well, and luciferase activity was measured 24 h later using Mitochondrial ToxGlo Assay (Promega) following manufacturer's recommendations. Carbonyl cyanide 3-chlorophenylhydrazone (CCCP) treatment at 20 μM for 90 min was used as positive control.

### FACS analysis ROS production

At 24 h post transfection, HeLa cells were incubated with 20 μM of the cell permeant 2′,7′–dichlorofluorescine diacetate (DCFDA) reagent for 1 h at 37 °C, following manufacturer's recommendations (DCFDA Cellular ROS Detection Assay Kit, Abcam). Cells were the washed twice in PBS, harvested, and analyzed by flowcytometry. Oxidative stress inducer tertiary-butyl hydroperoxide (TBHP) treatment was performed at a concentration of 100 μM for 30 min before DCFDA staining as positive control. Samples were analyzed on a MACSQuant Analyzer (Miltenyi Biotec) and data were processed with FlowJo software (Tree Star Inc, version 8.7.1).

### FACS analysis of inner mitochondrial membrane potential (Δψm)

Twenty-four hours posttransfection, the loss of inner mitochondrial membrane potential (Δψm) was evaluated in A3A-GFP transfected HeLa cells, using MitoStatus red (BD Pharmingen) Δψm-sensitive fluorescent dye. 70% confluent transfected HeLa cells were incubated with 100 nM MitoStatus Red in cell media for 20 min in the dark. FCCP (Carbonyl cyanide 4-(trifluoromethoxy)-phenylhydrazone) a mitochondrial oxidative phosphorylation uncoupler was used a positive control and incubated at a final concentration of 10 μM in culture media for 10 min at 37 °C before staining. After staining, cells were washed two times with PBS and harvested using trypsin. Cell pellet were washed two times in PBS and analyzed on a MACSQuant Analyzer (Miltenyi Biotec) and data were processed with FlowJo software (Tree Star Inc, version 8.7.1).

### FACS analysis of double-strand breaks

At 48 h after transfection, cells were washed with PBS, fixed in 2 to 4% ice-cold paraformaldehyde (Electron Microscopy Sciences) for 10 min, and permeabilized in 90% ice-cold methanol (Sigma) for 30 min. After washing with PBS, cells were incubated with 1:100 diluted mouse Alexa Fluor 647-conjugated anti-V5 antibody (clone SV5-Pk1, Biorad) and 1:50 Alexa Fluor 488-conjugated rabbit monoclonal anti-γH2AX (Ser139, 20E3) antibody (#9719, Cell Signaling Technology) in PBS-BSA 0.5% for 2 h. After PBS washings, DNA double-strand breaks were analyzed on a MACSQuant Analyzer (Miltenyi Biotec) and data were processed with FlowJo software (Tree Star Inc, version 8.7.1).

### FACS analysis of apoptosis

Transfected HeLa cells were harvested, counted, washed twice in PBS and 1 × 10^6^ cells were stained for 30 min with 1 μl 780eFluor of Fixable Viability Dye (eBioscience) in 1 ml PBS. For Z-VAD-FKM experiments, culture media was removed 6 h after transfection and replaced with 20 μM Z-VAD-FKM (Sigma) containing complete media. After PBS washing, cells were stained with 10 μl Annexin V-V450 (BD Biosciences, 560506) in 200 μl 1× binding buffer for 15 min. After washing, cells were fixed in 2% ice-cold paraformaldehyde (Electron Microscopy Sciences) for 15 min and permeabilized in 90% ice-cold methanol (Sigma-Aldrich) for 30 min. V5 staining was then performed using mouse anti-V5 Alexa-Fluor 647 conjugated antibody (clone SV5-Pk1, Biorad) and stained samples were acquired on a MACSQuant Analyzer (Miltenyi Biotec). Data were analyzed with FlowJo software (Tree Star Inc). Treatment with 100 μM etoposide (Sigma) in dimethylsulfoxide for 12 h was used as positive control.

## Data availability

Data sharing is not applicable to this article as no data libraries were generated. The communication author will accommodate requests of relevant materials.

## Supporting information

This article contains supporting information.

## Conflict of interest

The authors declare that they have no conflicts of interest with the contents of this article.
