# Supplementary material for: Frame-shifted APOBEC3A encodes two alternative proapoptotic proteins that target the mitochondrial network
Source: J Biol Chem. 2021 Aug 14;297(3):101081. doi: 10.1016/j.jbc.2021.101081 (PMC8424220; doi:10.1016/j.jbc.2021.101081)
Supplement: Figures S1–S14; Tables S1 and S2 [file mmc1.pdf]

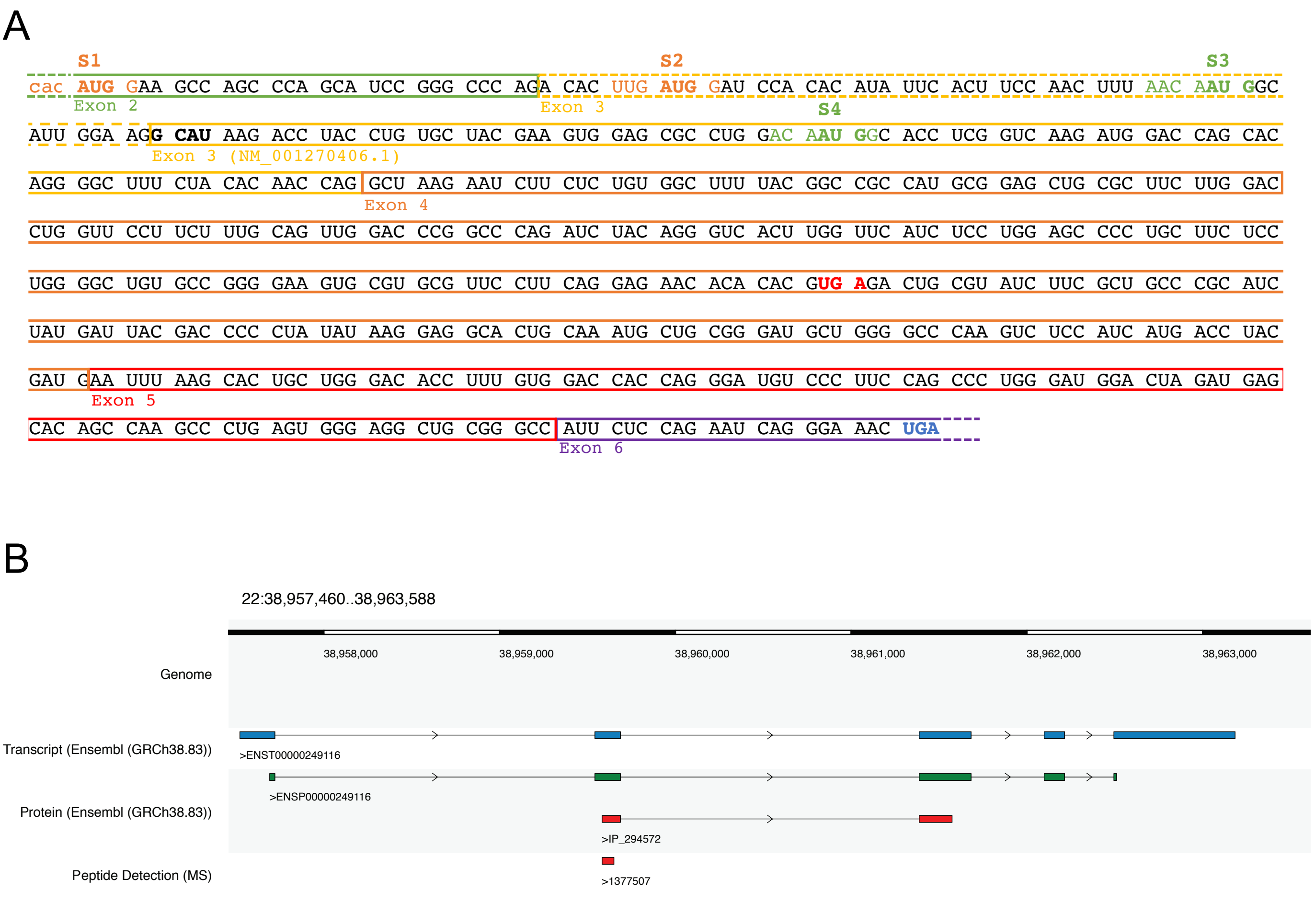

Study		peptide	match count?
BioPlex_2.0 http://bioplex.hms.harvard.edu/ *PMID: 28514442*	1	MALEGIRPTCATKWSAWTMAPR	1

Supporting Information Figure S1: Alternative protein expression from A3A transcript.

A) Annotated A3A mRNA sequence (NM_145699.4). Exons are represented boxed. The 5' portion of exon 3 represented in discontinued lines correspond nucleotides absent from A3A alternative transcript 3 (NM_001270406.1). Translation initiating codons S1, S2, S3 and S4 are represented in bold. Good Kozaks are represented in green, adequate in orange. A3A UGA stop codon is represented in blue, A3Alt UGA stop codon is represented in red. **B)** OpenProt *in silico* prediction of A3Alt-L (IP_294572, in red) expression from A3A transcript (ENST00000249116). Mass Spectrometry evidence of A3Alt-L derived peptide "MALEGIRPTCATKWSAWTMAPR" (1377507) from BioPlex 2.0 interactome study (PMID: 28514442). **C)** OpenProt *in silico* prediction of A3Alt-S (IP_294575, in red) expression from A3A alternative transcript 3 (NM_001270406.1).

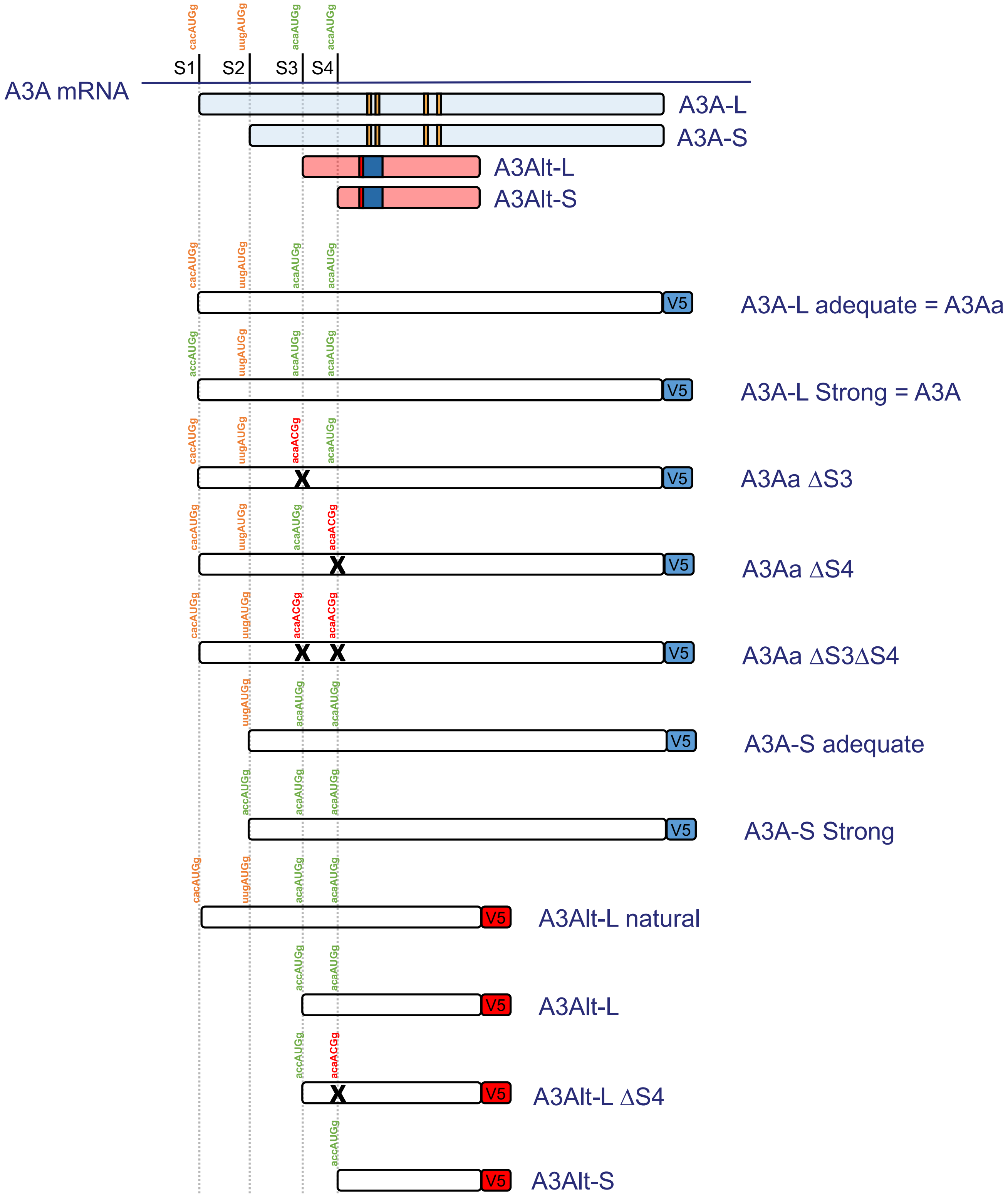

Supporting Information Figure S2: Schematic representation of A3A and A3Alt V5 tagged constructs. Strong, adequate or null Kozak context sequences are represented in green, orange and red respectively.V5 tag in A3A classical cytidine deaminase reading frame are represented in blue, V5 tag in the +1 A3Alt reading frame are represented in red. Start codons invalidated by site directed mutagenesis are crossed out in black.

A

A3Alt-L

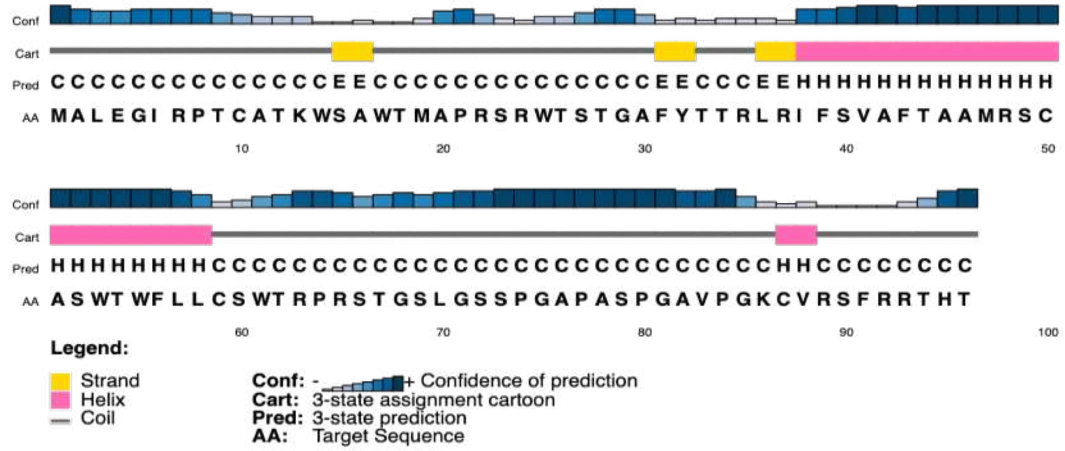

A3Alt-S

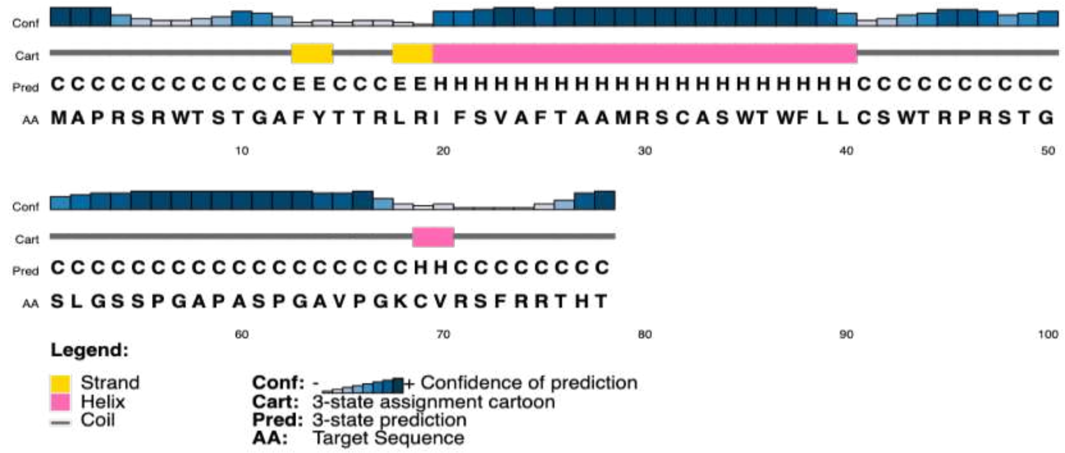

B

A3Alt-L

Localization	Mitochondrion	Peroxisome	Cell membrane	Endoplasmic reticulum	Plastid	Cytoplasm	Nucleus	Extracellular	Golgi apparatus	Lysosome/Vacuole
Likelihood	0.9159	0.0249	0.0178	0.0154	0.0087	0.0064	0.0052	0.0025	0.0023	0.0009

A3Alt-S

Localization	Mitochondrion	Cytoplasm	Extracellular	Nucleus	Peroxisome	Cell membrane	Golgi apparatus	Plastid	Lysosome/Vacuole	Endoplasmic reticulum
Likelihood	0.8866	0.0384	0.0239	0.0222	0.0155	0.0084	0.0031	0.0008	0.0007	0.0005

C

Targeting peptide prediction

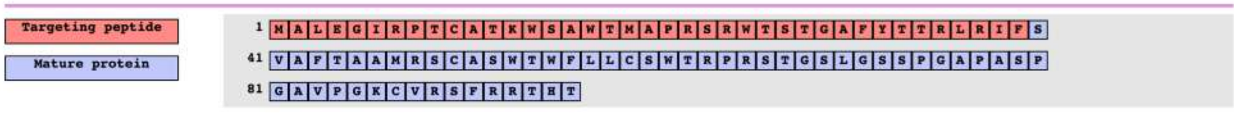

Motif occurence at cleavage site

The list of motifs that match the region around the predicted cleavage site.

Motif name	Sequence match	Start	End
R3b	RIFS	37	40
R3a	RIFS	37	40

Supporting Information Figure S3 : *In silico* analysis of A3Alt proteins.
A) A3Alt-L and A3Alt-S domain prediction using PSIPRED 4.0 software. Transmembrane helix domain is represented in pink. **B)** Prediction of A3Alt proteins localization using DeepLoc software. **C)** Mitochondrial targeting peptide prediction using Ttpred2 software. RIFS residues in A3Alt proteins are compatible with R3a/R3b cleavage site by MPP, with R3a motif being Rx[FLY] / [SA], and R3b being Rx[FLY] / x where « / » indicates cleavage sites, « x » any residue and the residues in brackets correspond to alternative choices.

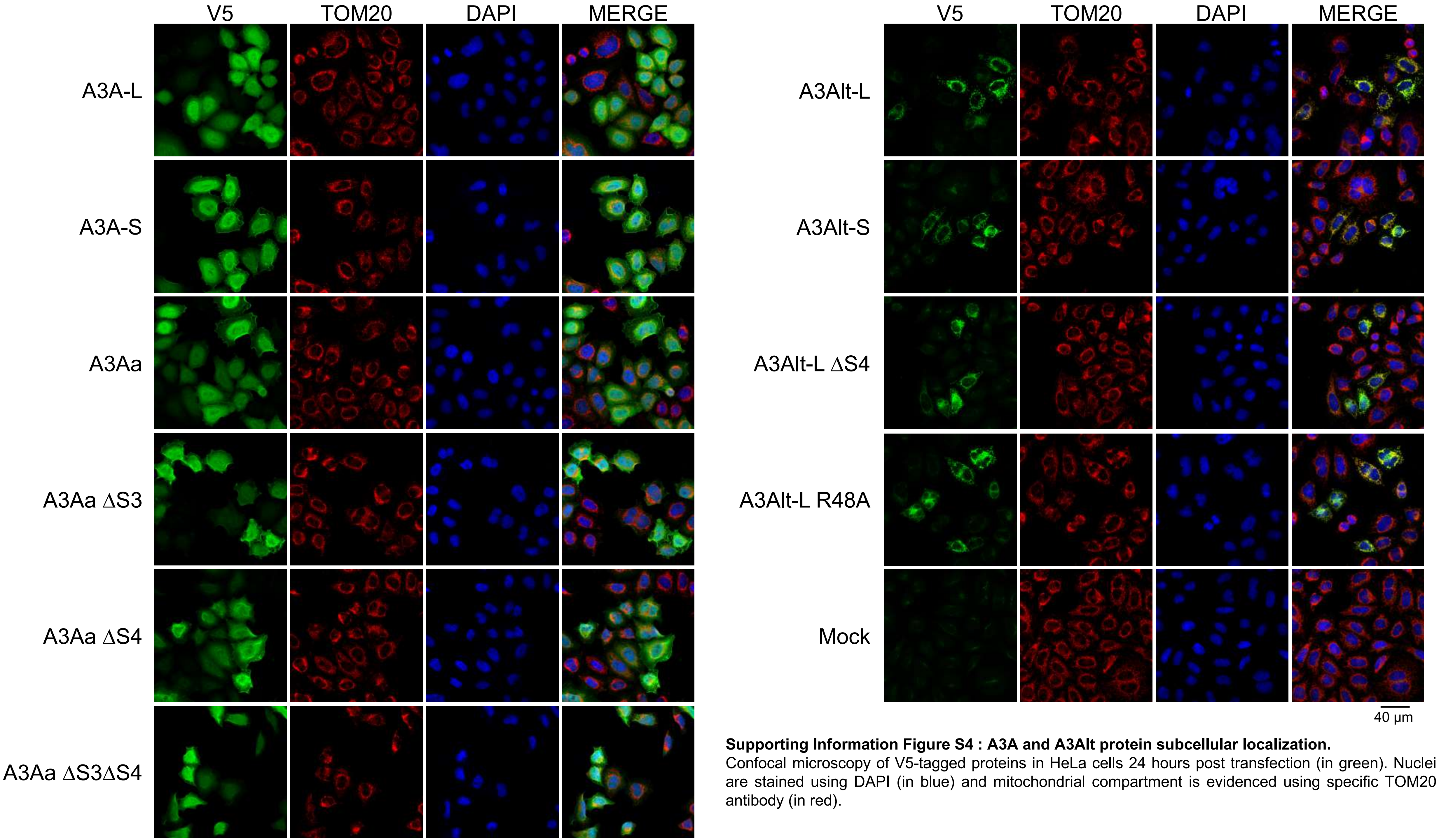

Supporting Information Figure S4 : A3A and A3Alt protein subcellular localization.
Confocal microscopy of V5-tagged proteins in HeLa cells 24 hours post transfection (in green). Nuclei are stained using DAPI (in blue) and mitochondrial compartment is evidenced using specific TOM20 antibody (in red).

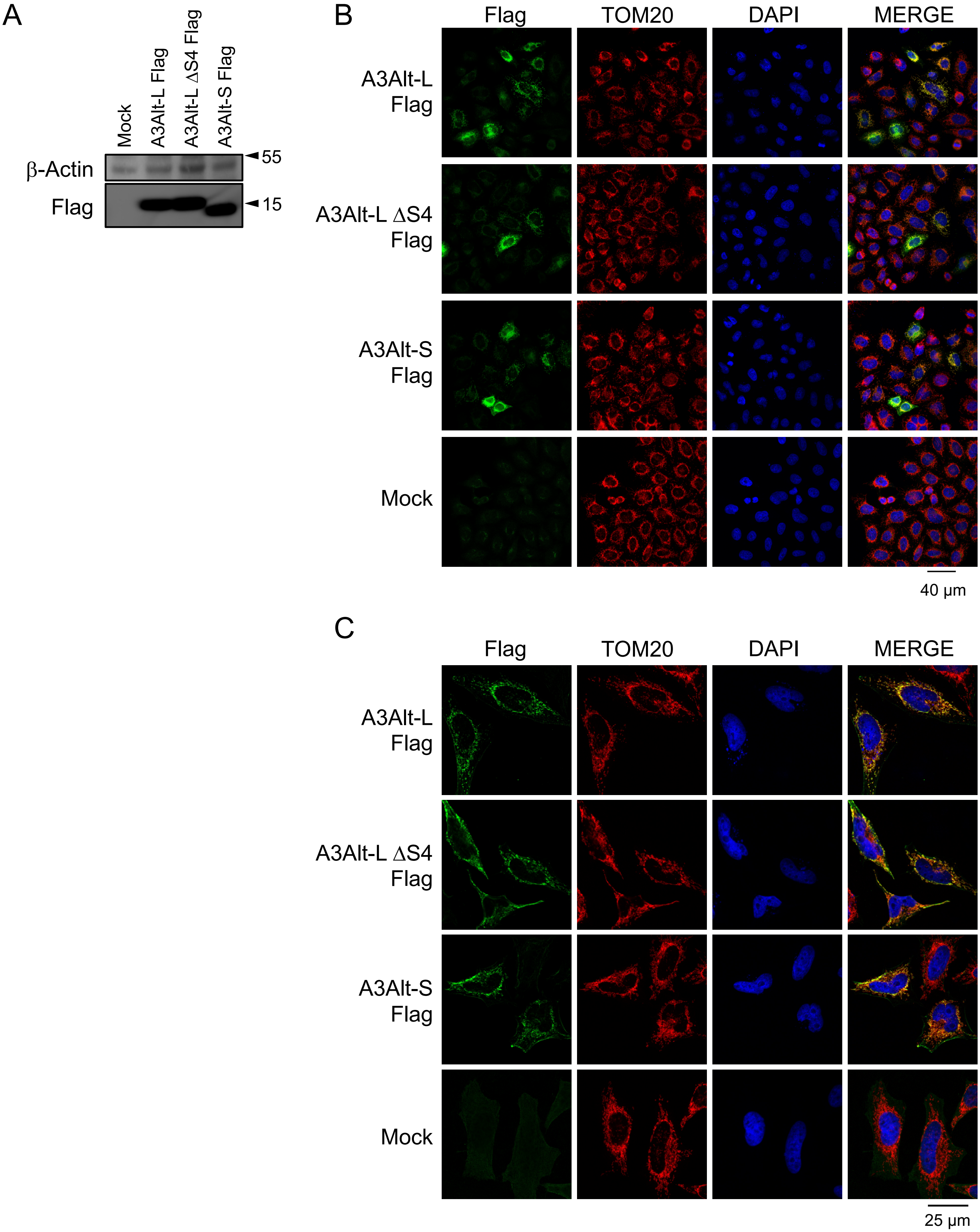

Supporting Information Figure S5 : Flag-tagged A3Alt proteins expression.
A) Western-blot analysis of Flag-tagged A3Alt proteins in HeLa cells 24 hours post transfection. β-Actin was used as loading control. **B)** and **C)** Confocal microscopy of Flag-tagged proteins expression in HeLa cells 24 hours post transfection (in green). Nuclei are stained using DAPI (in blue) and mitochondrial compartment is evidenced using specific TOM20 antibody (in red).

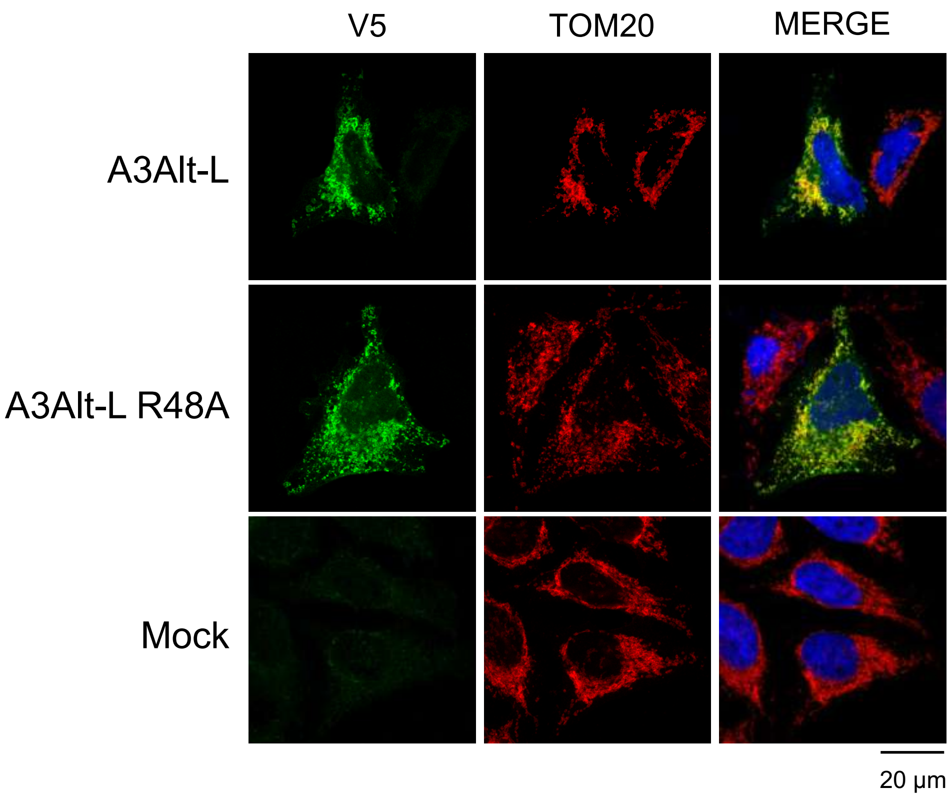

Supporting Information Figure S6 : A3Alt-L and A3Alt-L R48A mutant share similar subcellular localization. Confocal microscopy of V5 tagged A3Alt-L and A3Alt-L R48A mutant expression in HeLa cells 24 hours post transfection (in green). Nuclei are stained using DAPI (in blue) and mitochondrial compartment is evidenced using specific TOM20 antibody (in red).

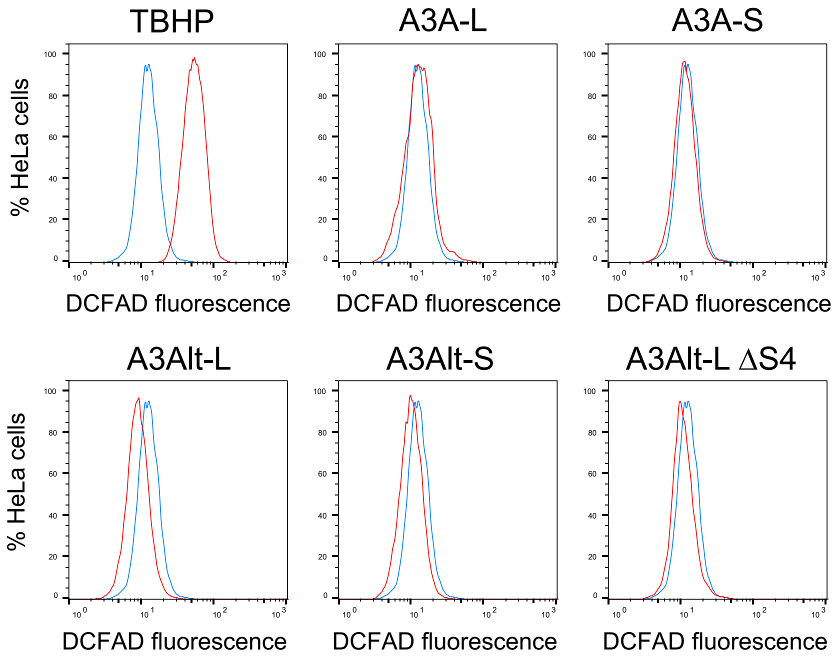

Supporting Information Figure S7 : Reactive Oxygen Species (ROS) levels in A3A and A3Alt expressing Hela cells.
Histogram plots of flow cytometry analysis of of DCFAD staining of A3A and A3Alt transfected Hela cells 24 hours after transfection. Blue histograms represent DCFAD staining (X axis) of Mock transfected cells, red histograms the DCFAD staining of A3A and A3Alt conditions. Oxidative stress inducer TBHP (100 µM) was used as positive control for ROS production.

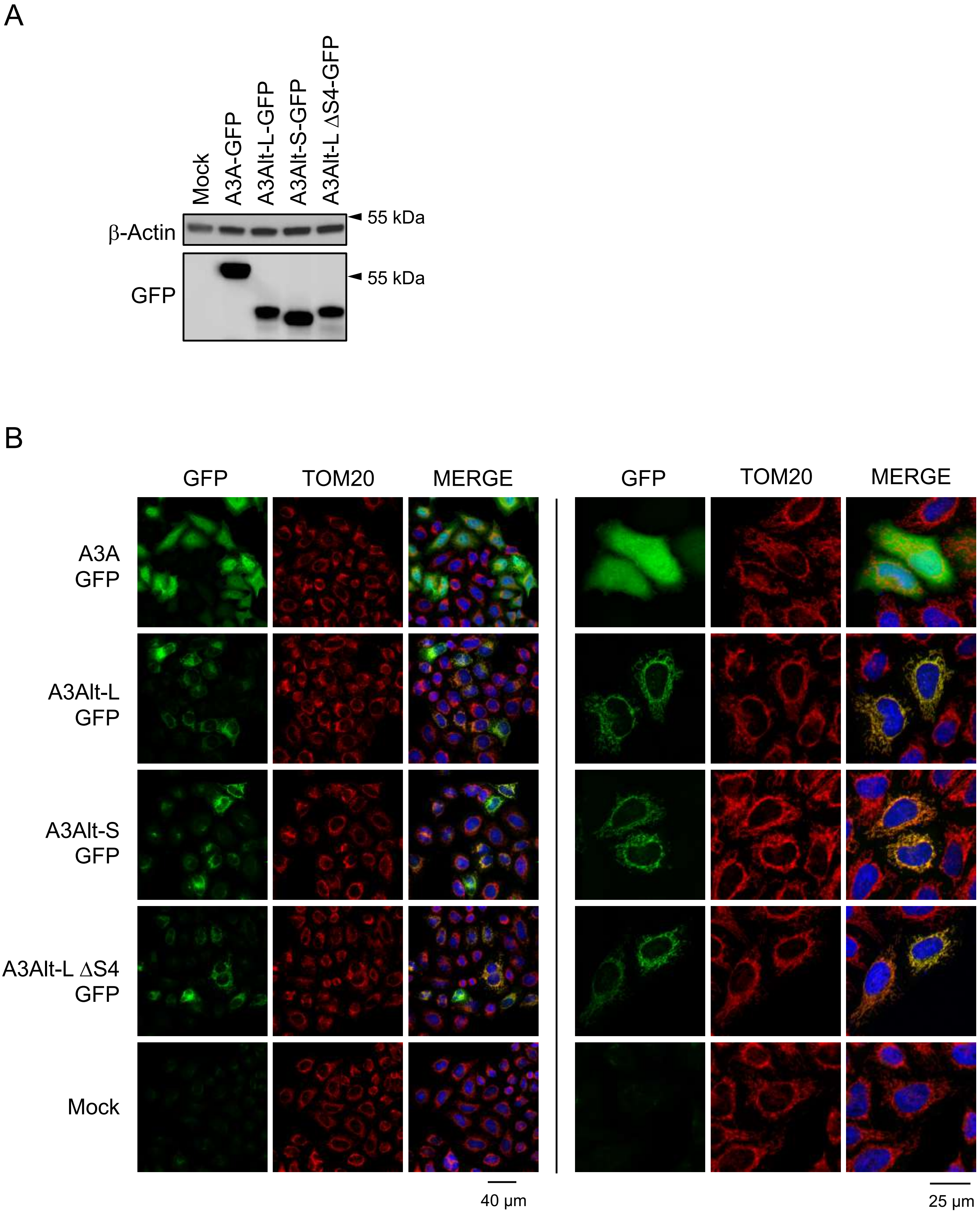

Supporting Information Figure S8 : GFP-tagged A3A and A3Alt proteins expression.
A) Western-blot analysis of GFP-tagged A3A and A3Alt proteins in HeLa cells 24 hours post transfection. β-Actin was used as loading control. **B)** Confocal microscopy of GFP-tagged proteins in HeLa cells 24 hours post transfection (in green). Nuclei are stained using DAPI (in blue) and mitochondrial compartment is evidenced using specific TOM20 antibody (in red).

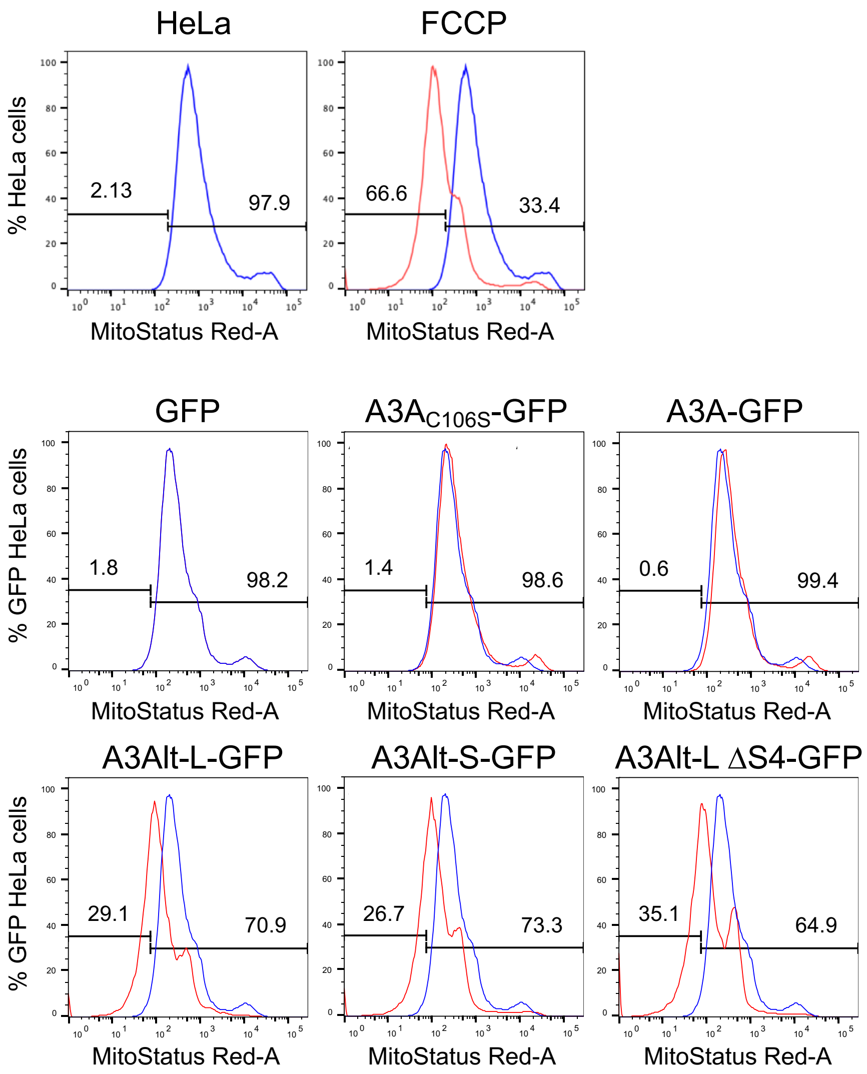

Supporting Information Figure S9 : Flow cytometry analysis of mitochondrial membrane potential depolarisation (Δψm).

Flow cytometry plots of mitochondrial membrane potential measured with MitoStatus Red ψm-sensitive dye (X axis). FCCP oxidative phosphorylation uncoupler treated Hela cells are used as positive control. Blue histogram represent mitochondrial membrane potential of control Hela cells (top panel) or GFP transfected control cells (bottom panel), red histogram the mitochondrial membrane potential of FCCP treated HeLa cells (top panel), or A3A and A3Alt transfected cells (bottom panel). Numbers on the left of blue histograms correspond to the percentage of cells displaying a loss of mitochondrial membrane potential compared to control condition, numbers on the right the number of cells displaying a mitochondrial membrane potential similar to control condition.

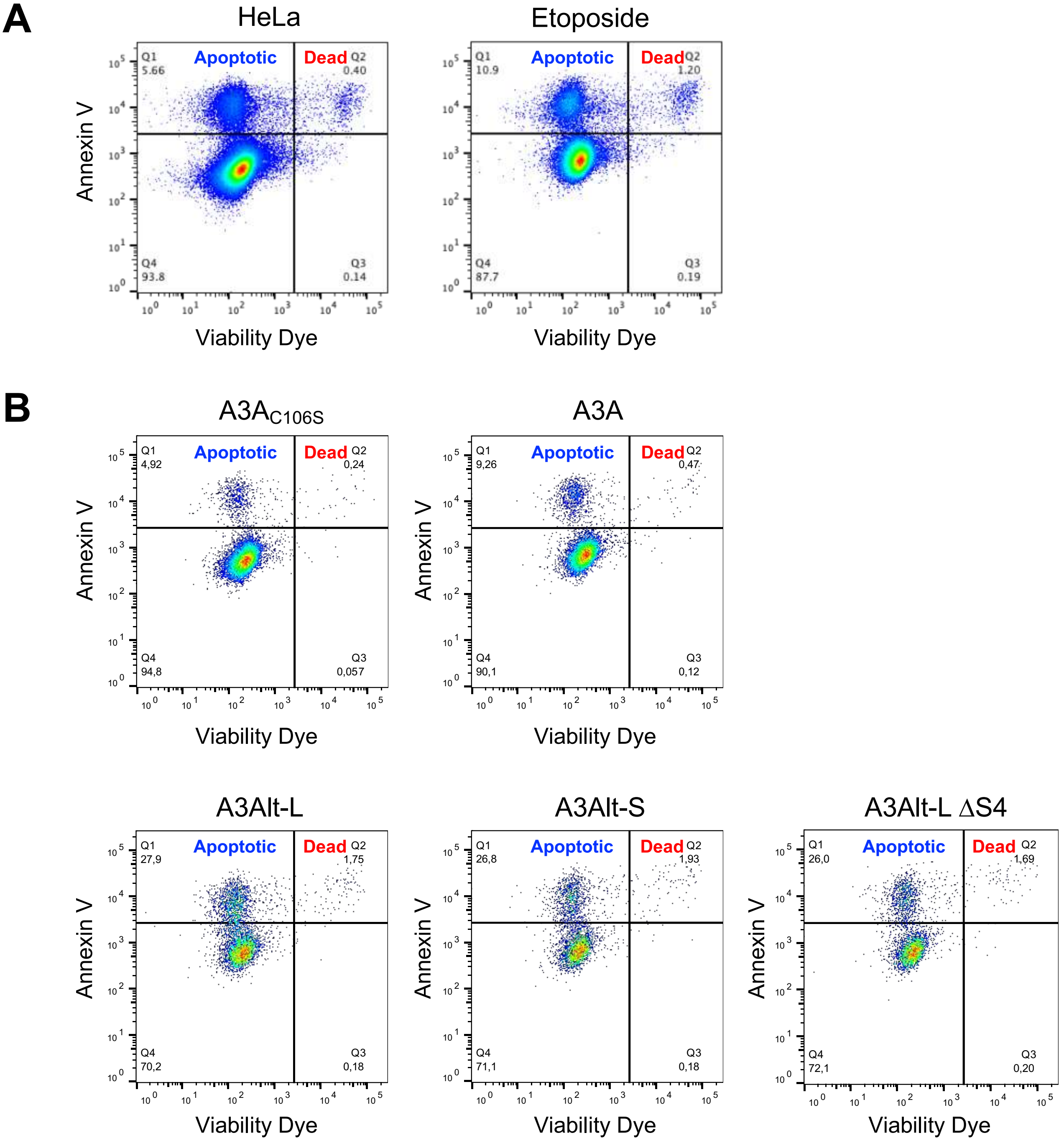

Supporting Information Figure S10 : Flow cytometry plots of apoptosis.

A) Annexin V and viability dye staining of non treated and 100 µM Etoposide treated HeLa cells. **B)** Annexin V and viability dye staining in V5 after A3A and A3Alt transfection of HeLa HeLa cells. Bottom left quadrant (Annexin V -, Viability dye –) corresponds to normal cells, top left quadrant (Annexin V +, Viability dye –) corresponds to early apoptotic cells; top right quadrant (Annexin V +, Viability dye +) correspond to late apoptotic cells. Values correspond to the percentage of cells in those quadrants.

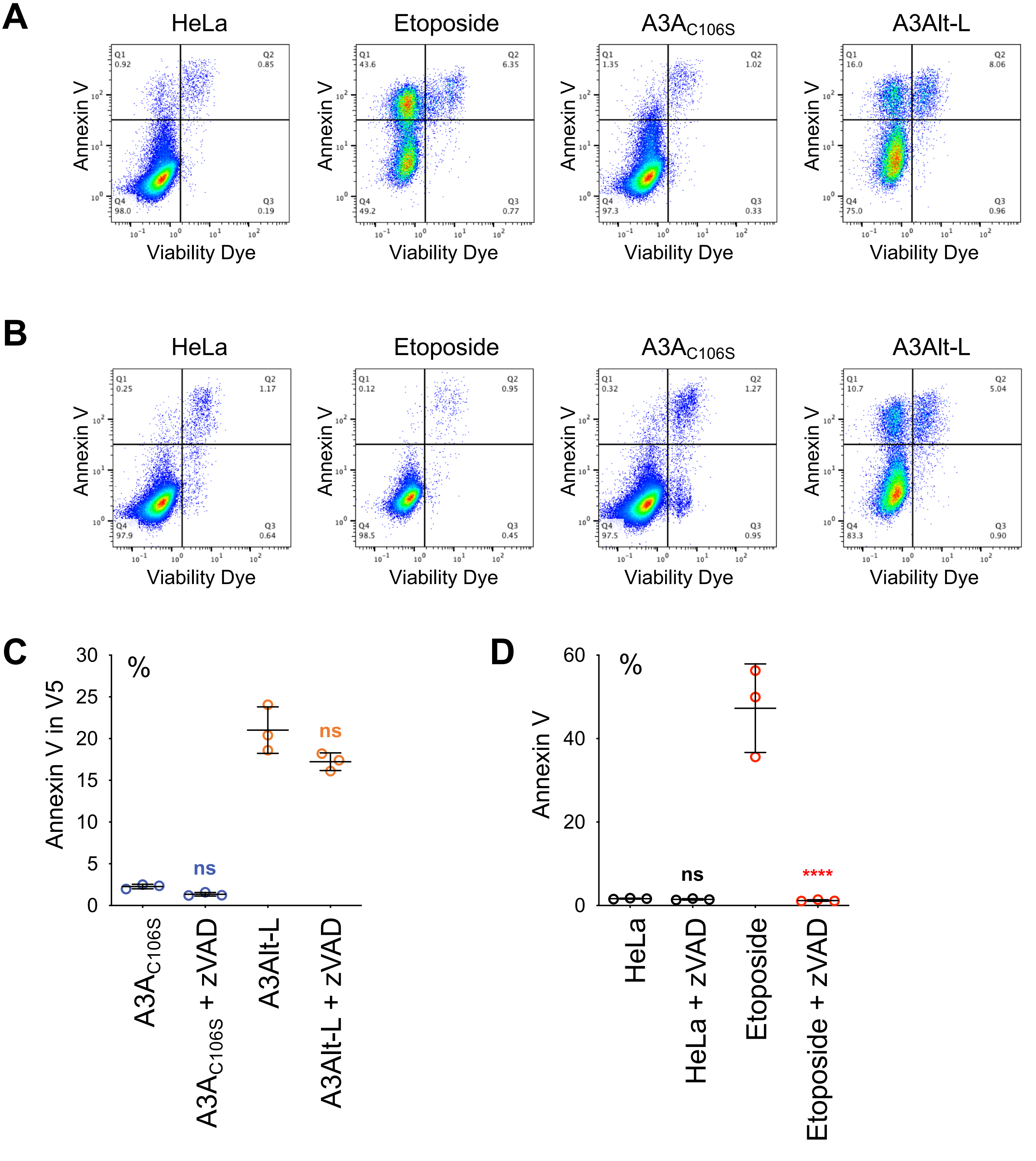

Supporting Information Figure S11 : Flow cytometry plots of apoptosis with or without Z-VAD-FKM pan-caspase inhibitor.

A) Annexin V and Viability Dye staining of non treated, 100 µM Etoposide treated HeLa cells and A3AC106S / A3Alt-L transfected HeLa cells in absence of Z-VAD-FKM pan-caspase inhibitor . **B)** Annexin V and Viability Dye staining of non treated, 100 µM Etoposide treated HeLa cells and A3AC106S / A3Alt-L transfected HeLa cells in presence of 20 µM Z-VAD-FKM pan-caspase inhibitor. Bottom left quadrant (Annexin V -, Viability Dye –) corresponds to normal cells, top left quadrant (Annexin V +, Viability Dye –) corresponds to early apoptotic cells; top right quadrant (Annexin V +, Viability Dye +) correspond to late apoptotic cells. Values correspond to the percentage of cells in those quadrants. **C)** Flow cytometry analysis of Annexin V staining in transfected HeLa cells. Error bars represent standard deviation from three independent transfections. Differences compared to A3AC106S catalytic mutants are represented blue, and differences compared to A3Alt-L are represented in orange. **D)** Flow cytometry analysis of Annexin V staining in HeLa cells. Error bars represent standard deviation from three independent experiments. Difference compared to non Z-VAD-FKM treated HeLa cells are represented black, and difference compared to non Z-VAD-FKM treated Etoposide treated HeLa cells are represented in orange. Differences were calculated using unpaired two-tailed Student's t-test (ns : non significative, **** : $p < 0.0001$).

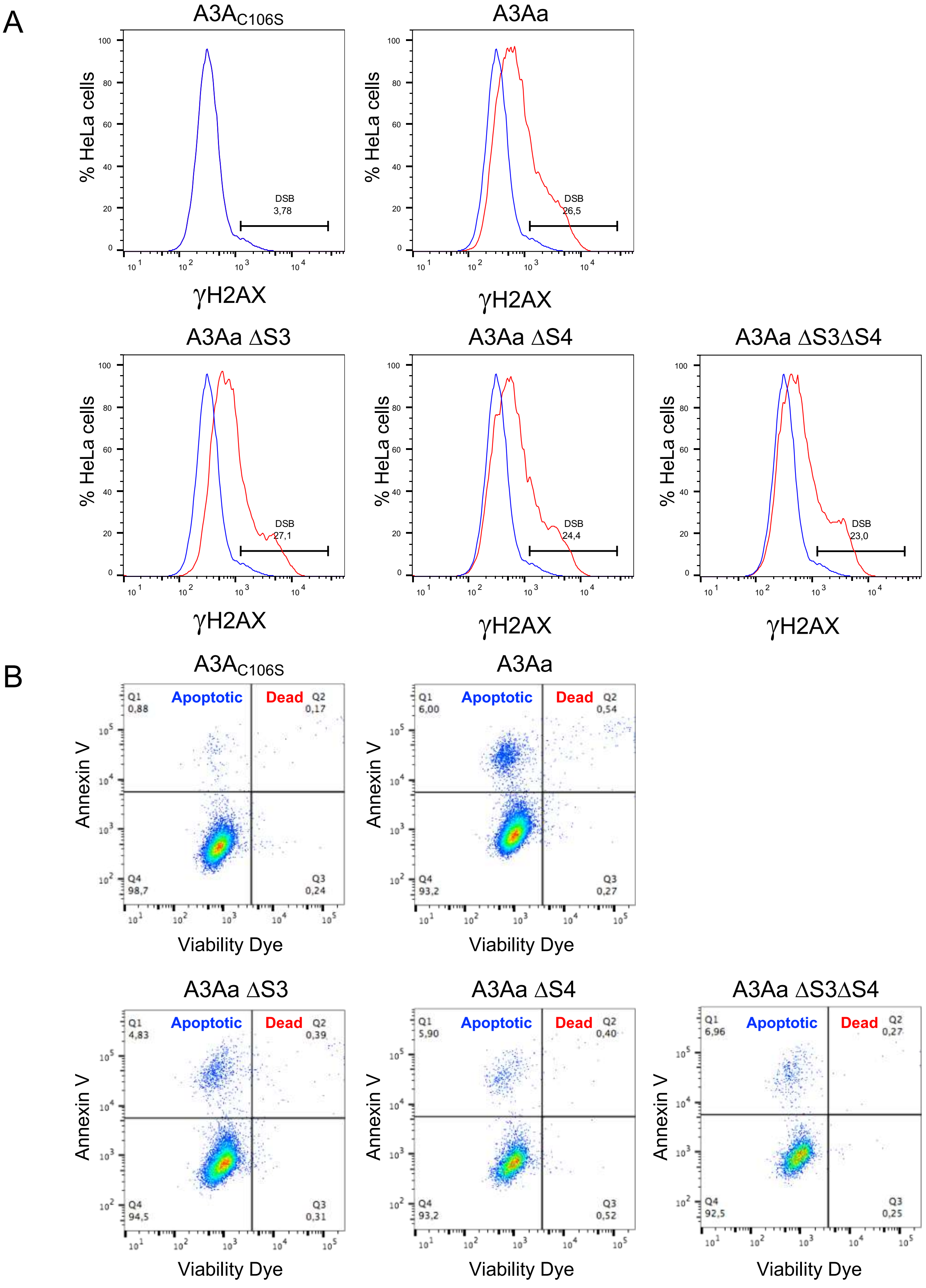

Supporting Information Figure S12 : Flow cytometry analysis of double strand breaks and apoptosis induced by A3Aa and A3Aa mutants.

A) Flow cytometry analysis of γH2AX-positive HeLa cells gated on V5-positive cells after A3Aa and A3A mutants transfections after 48 hours. **B)** Annexin V and viability dye staining in V5 after A3Aa and A3A mutants transfection of HeLa HeLa cells. Bottom left quadrant (Annexin V -, Viability dye –) corresponds to normal cells, top left quadrant (Annexin V +, Viability dye –) corresponds to early apoptotic cells; top right quadrant (Annexin V +, Viability dye +) correspond to late apoptotic cells. Values correspond to the percentage of cells in those quadrants.

A

```
                       S3                S4
Human           ---MALEGIRPTCATKWSAWTMAPRSRWTSTGAFYTTRLRIFSVAFTAAMRSCASWTWFLLCSWTRPRSTGSLGSSPGAPASPGAVPGKCVRSFRRTHT
Homo sapiens

Orangutan       ---MALDGIRPICAMRWSAWTMAPGSRWTSTGAFYTTRLGILSMAWTAAMRSCASWACFLIGSWTRPRSTGSLGSSPGAPASPGAVPGKCVHSFRRTHT
Pongo Abelii

Gorilla         ---MALEGIRPTCATKWSAWTMAPGSRWTSTGAFYTTRLRIFSVAFTAAMRSCASWTWFLLCSWTRPRSTGSLGSSPGAPASPGAVPGKCVRSFRRTHT
Gorilla gorilla

Bonobo          ---------------------MAPRSRWTSTGAFYTTRLRIFSVAFTAAMRSCASWTWFLLCSWTRPRSTGSLGSSPGAPASPGAVPGKCVRSFRRTHT
Pan paniscus

Gibbon          ---MALEGIRPTCATRWSTWTMAHGSRWTSTGALYTTRLRIFSLAFTAAMRSCASWTWFLLCSWTWPRPTGSLGSSPGAPASPGAVLSECVRSFRRTHT
Hylobates lar

Chimpanzee      ---------------------MAPRSRWTSTGAFYTTRLRIFSVAFTAAMRSCASWTWFLLCSWTRPRSTGSLGSSPGAPASPGAVPGKCVRSFRRTHT
Pan troglodytes

Rhesus Macaque  MTFRSVDGTRPTCATRWSAWTMAPGSRWTSAGAFYATRLRMFPVVITAATWSCASCVRFLLGSWTRPRHTGSLGSSPGAPASGGAVPGKCVCSFRRTNT
Macaca mulatta
```

B

	S1	Kozak context	S2	Kozak context	S3	Kozak context	S4	Kozak context
Human	CACAUGG	Adequate	UUGAUGG	Adequate	ACAAUGG	Strong	ACAAUGG	Strong
Orangutan	CACAUGG	Adequate	UUGAUGG	Adequate	ACAAUGG	Strong	ACAAUGG	Strong
Gorilla	CACGTGG	Null	UUCAUGG	Adequate	ACAAUGG	Strong	ACAAUGG	Strong
Bonobo	CACACGG	Null	UUGAUGG	Adequate	ACAACGG	Null	ACAAUGG	Strong
Gibbon	CACACGG	Null	UUGAUGG	Adequate	ACAAUGG	Strong	ACAAUGG	Strong
Chimpanzee	CACACGG	Null	UUGAUGG	Adequate	ACAACGG	Null	ACAAUGG	Strong
Rhesus	CACAUGG	Adequate	UUGAUGG	Adequate	ACAAUGA	Adequate	ACAAUGG	Strong

Supporting Information Figure S13 : A3A translational organisation is shared among primates.
A) A3Alt proteins from various primates with S3 and S4 start codons represented in green. Difference from human A3Alt protein sequences are depicted in red. **B)** Kozak sequence contexts surrounding the A3A S1, S2, S3 and S4 initiation codons from various primates transcripts.

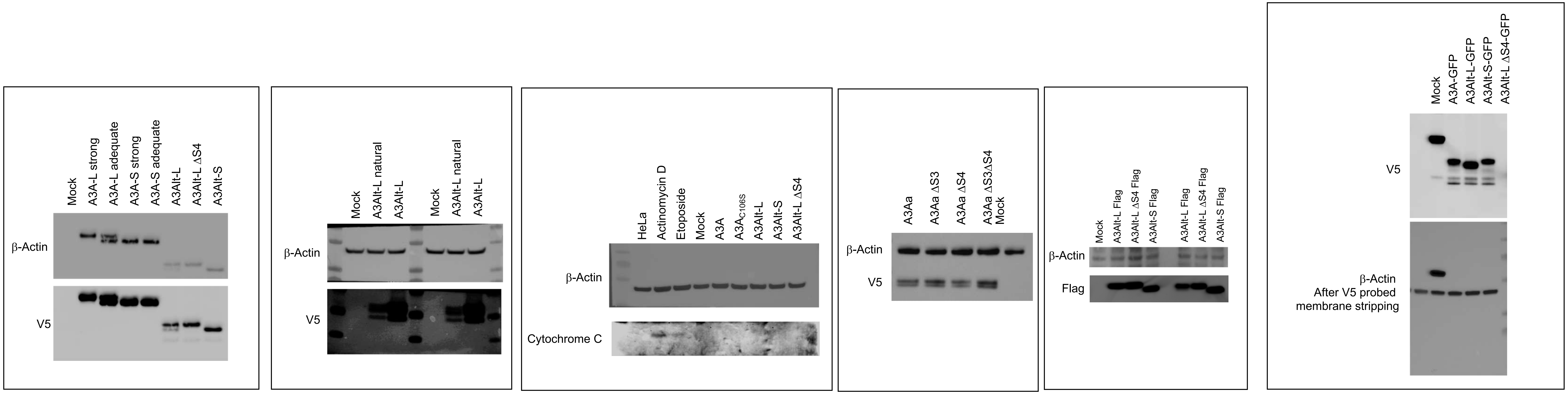

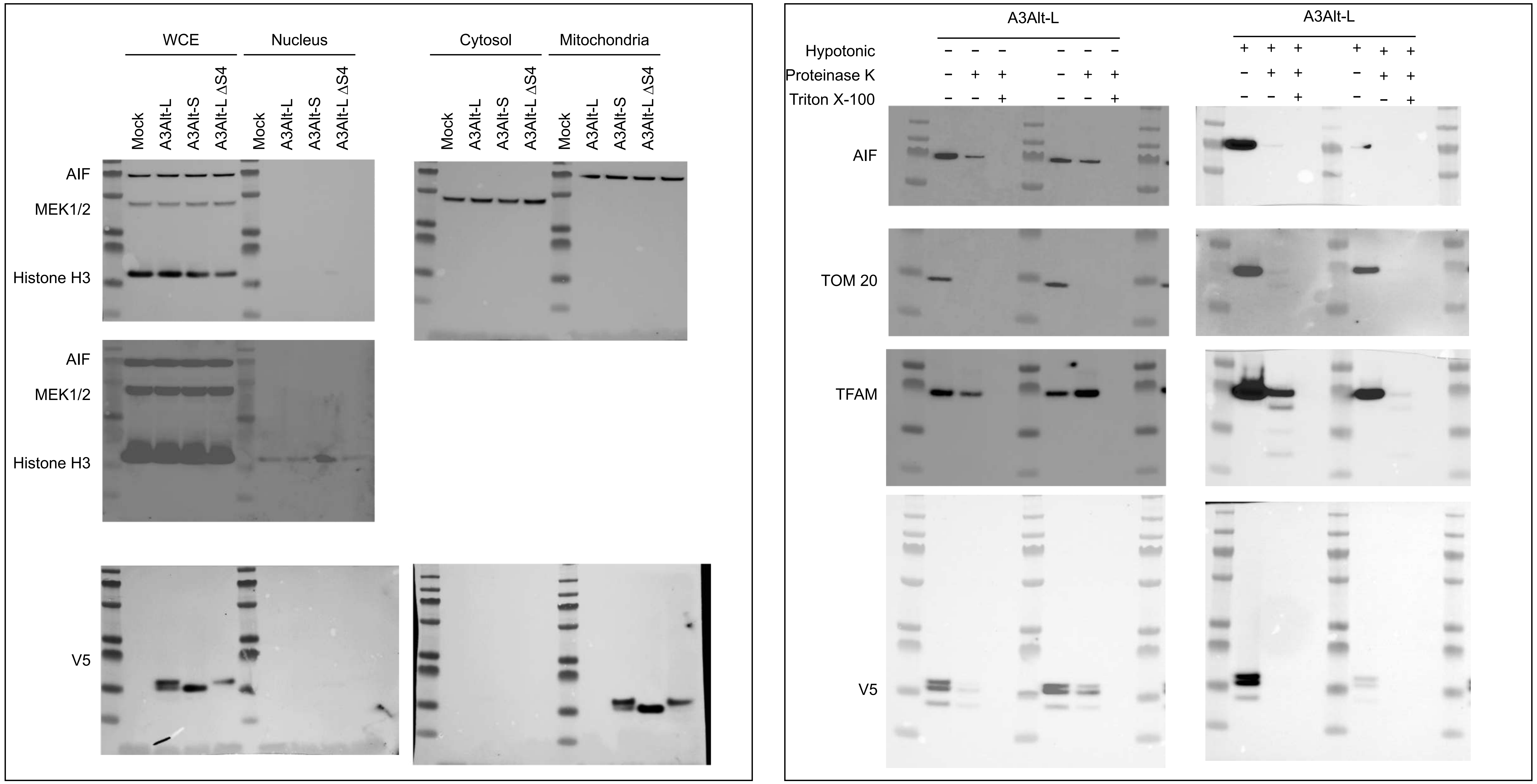

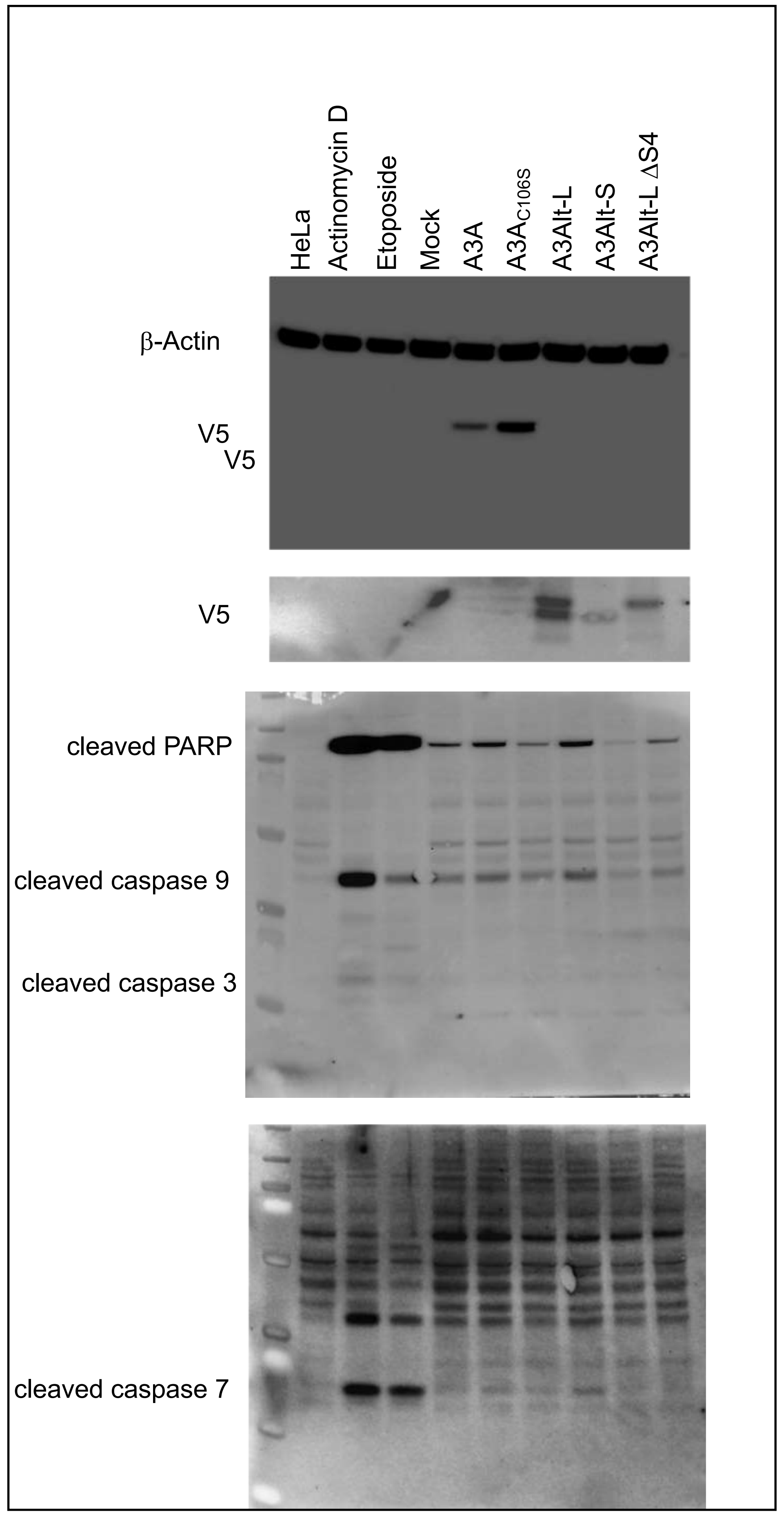

Supporting Information Figure S14: Uncropped versions of presented western blots.

Plasmid	Matrix	Primers
A3Alt-L	pcDNA3.1-A3A	For : 5′-CACC**ATG**GCATTGGAAGGCATAAGACCTACCTG<br>Rev : 5′-CGTGTGTGTTCTCCTGAAGG
A3Alt-S	pcDNA3.1-A3A	For : 5′-CACC**ATG**GCACCTCGGTCAAGATGGACCAGCACAGG<br>Rev : 5′-CGTGTGTGTTCTCCTGAAGG
A3Alt natural	A3A adequate A3Aa	For : 5′- CACCGAGAAGGGACAAGCAC**ATG**GAAGCCAGCCCAGCATCCGGGCCCAGACACTTG<br>Rev : 5′-CGTGTGTGTTCTCCTGAAGG
A3Alt-L Flag<br>A3Alt-L ΔS4 Flag	pcDNA3.1-A3A<br>pcDNA3.1-A3Alt-L ΔS4	For : 5′- CACC**ATG**GCATTGGAAGGCATAAGACCTACCTG<br>Rev : 5′- CTACTTGTCATCGTCGTCCTTGTAGTCCGTGTGTGTTCTCCTGAAGGAACGCACGCAC
A3Alt-S Flag	pcDNA3.1-A3A	For : 5′- CACC**ATG**GCACCTCGGTCAAGATGGACCAGCACAGG<br>Rev : 5′- CTACTTGTCATCGTCGTCCTTGTAGTCCGTGTGTGTTCTCCTGAAGGAACGCACGCAC
S1$^+$ S2$^+$ Luc	pcDNA3.1-A3A	For : 5′- CACCGAGAAGGGACAAGCAC**ATG**GAAGCCAGCCCAGCATCCGGGCCCAGACACTTG<br>Rev : 5′- CTTCTTAATGTTTTTGGCATCTTCCATCAAGTGTCTGGGCCCGGATGC
	pcDNA3.1-Luc	For : 5′- GCATCCGGGCCCAGACACTTG**ATG**GAAGATGCCAAAAACATTAAGAAG<br>Rev : 5′- TTACACGGCGATCTTGCCGCCCTTC
S1$^+$ S2$^-$ Luc	pcDNA3.1-A3A	For : 5′- CACCGAGAAGGGACAAGCAC**ATG**GAAGCCAGCCCAGCATCCGGGCCCAGACACTTG<br>Rev : 5′- CTTCTTAATGTTTTTGGCATCTTCCAGCAAGTGTCTGGGCCCGGATGC
	pcDNA3.1-Luc	For : 5′- GCATCCGGGCCCAGACACTTG**CTG**GAAGATGCCAAAAACATTAAGAAG<br>Rev : 5′- TTACACGGCGATCTTGCCGCCCTTC
S1$^-$ S2$^+$ Luc	pcDNA3.1-A3A	For : 5′- CACCGAGAAGGGACAAGCAC**CTG**GAAGCCAGCCCAGCATCCGGGCCCAGACACTTG<br>Rev : 5′- CTTCTTAATGTTTTTGGCATCTTCCATCAAGTGTCTGGGCCCGGATGC
	pcDNA3.1-Luc	For : 5′- GCATCCGGGCCCAGACACTTG**ATG**GAAGATGCCAAAAACATTAAGAAG<br>Rev : 5′- TTACACGGCGATCTTGCCGCCCTTC
S1$^-$ S2$^-$ Luc	pcDNA3.1-A3A	For : 5′- CACCGAGAAGGGACAAGCAC**CTG**GAAGCCAGCCCAGCATCCGGGCCCAGACACTTG<br>Rev : 5′- CTTCTTAATGTTTTTGGCATCTTCCAGCAAGTGTCTGGGCCCGGATGC
	pcDNA3.1-Luc	For : 5′- GCATCCGGGCCCAGACACTTG**CTG**GAAGATGCCAAAAACATTAAGAAG<br>Rev : 5′- TTACACGGCGATCTTGCCGCCCTTC
S1$^+$ S2$^+$ Luc	pcDNA3.1-A3A	For : 5′- CACCGAGAAGGGACAAGCAC**ATG**GAAGCCAGCCCAGCATCCGGGCCCAGACACTTG<br>Rev : 5′- CTTCTTAATGTTTTTGGCATCTTCCATCAAGTGTCTGGGCCCGGATGC
	pcDNA3.1-Luc	For : 5′- GCATCCGGGCCCAGACACTTG**ATG**GAAGATGCCAAAAACATTAAGAAG<br>Rev : 5′- TTACACGGCGATCTTGCCGCCCTTC
S3$^+$ S4$^+$ Luc<br>S3$^-$ S4$^+$ Luc	pcDNA3.1-A3A<br>pcDNA3.1-A3Aa ΔS3	For : 5′- CACCGAGAAGGGACAAGCAC**ATG**GAAGCCAGCCCAGCATCCGGGCCCAGACACTTG<br>Rev : 5′- CTTCTTAATGTTTTTGGCATCTTCCATTGTCCAGGCGCTCCACTTCGT
	pcDNA3.1-Luc	For : 5′- ACGAAGTGGAGCGCCTGGACA**ATG**GAAGATGCCAAAAACATTAAGAAG<br>Rev : 5′- TTACACGGCGATCTTGCCGCCCTTC
S3$^+$ S4$^-$ Luc<br>S3$^-$ S4$^-$ Luc	pcDNA3.1-A3A<br>pcDNA3.1-A3Aa ΔS3	For : 5′- CACCGAGAAGGGACAAGCAC**ATG**GAAGCCAGCCCAGCATCCGGGCCCAGACACTTG<br>Rev : 5′- CTTCTTAATGTTTTTGGCATCTTCCAGTGTCCAGGCGCTCCACTTCGT
	pcDNA3.1-Luc	For : 5′- ACGAAGTGGAGCGCCTGGACA**CTG**GAAGATGCCAAAAACATTAAGAAG<br>Rev : 5′- TTACACGGCGATCTTGCCGCCCTTC

Supplementary table 1. Compendium of primers used for cloning.

**Plasmid**	**Matrix**	**Primers**
A3Aa ΔS3	pcDNA3.1-A3Aa	For : 5'- CTTCCAACTTTAACA**ACG**GCATTGGAAGGCATA<br>Rev : 5'- TATGCCTTCCAATGC**CGT**TGTTAAAGTTGGAAG
A3Alt-L ΔS4<br>A3Aa ΔS4<br>A3Aa ΔS3ΔS4	pcDNA3.1-A3Alt-L<br>pcDNA3.1-A3Aa<br>pcDNA3.1-A3Aa ΔS3	For : 5'- TGGAGCGCCTGGACA**ACG**GCACCTCGGTCAAGA<br>Rev : 5'- TCTTGACCGAGGTGC**CGT**TGTCCAGGCGCTCCA
A3Alt-L R48A	pcDNA3.1-A3Alt-L	For : 5'- TTTACGGCCGCCATG**GCG**AGCTGCGCTTCTTGG<br>Rev : 5'- CCAAGAAGCGCAGCT**CGC**CATGGCGGCCGTAAA

Supplementary table 2. Compendium of primers used for mutagenesis
